# A European Concern? Genetic Structure and Expansion of Golden Jackals (*Canis aureus*) in Europe and the Caucasus

**DOI:** 10.1371/journal.pone.0141236

**Published:** 2015-11-05

**Authors:** Robert Rutkowski, Miha Krofel, Giorgos Giannatos, Duško Ćirović, Peep Männil, Anatoliy M. Volokh, József Lanszki, Miklós Heltai, László Szabó, Ovidiu C. Banea, Eduard Yavruyan, Vahram Hayrapetyan, Natia Kopaliani, Anastasia Miliou, George A. Tryfonopoulos, Petros Lymberakis, Aleksandra Penezić, Giedrė Pakeltytė, Ewa Suchecka, Wiesław Bogdanowicz

**Affiliations:** 1 Museum and Institute of Zoology, Polish Academy of Sciences, Warszawa, Poland; 2 Wildlife Ecology Research Group, Department of Forestry, Biotechnical Faculty, University of Ljubljana, Ljubljana, Slovenia; 3 Department of Zoology - Marine Biology, School of Biology, University of Athens, Panepistimioupolis, Athens, Greece; 4 Institute of Zoology, Faculty of Biology, University of Belgrade, Belgrade, Serbia; 5 Estonian Environment Agency, Tartu, Estonia; 6 Tauride Agrotechnology State University, Melitopol’, Ukraine; 7 Department of Nature Conservation, University of Kaposvár, Kaposvár, Hungary; 8 Institute for Wildlife Conservation, Szent István University, Gödöllő, Hungary; 9 Ecology Department of Crispus NGO Sibiu, Sibiu, Romania; 10 Scientific Centre of Zoology and Hydroecology, National Academy of Sciences of Armenia, Yerevan, Armenia; 11 Stepanakert Branch of the Armenian National Agrarian University, Stepanakert, Armenia; 12 Institute of Ecology, Ilia State University, Tbilisi, Georgia; 13 Archipelagos Institute of Marine Conservation, Mesokampos, Pythagorio, Samos, Greece; 14 Management Body of Mount Parnon & Moustos Wetland, Arkadia, Astros, Greece; 15 Natural History Museum of Crete, University of Crete, Heraklion, Crete, Greece; 16 Kaunas T. Ivanauskas Zoological Museum, Kaunas, Lithuania; National Cheng-Kung University, TAIWAN

## Abstract

In the first continent-wide study of the golden jackal (*Canis aureus*), we characterised its population genetic structure and attempted to identify the origin of European populations. This provided a unique insight into genetic characteristics of a native carnivore population with rapid large-scale expansion. We analysed 15 microsatellite markers and a 406 base-pair fragment of the mitochondrial control region. Bayesian-based and principal components methods were applied to evaluate whether the geographical grouping of samples corresponded with genetic groups. Our analysis revealed low levels of genetic diversity, reflecting the unique history of the golden jackal among Europe’s native carnivores. The results suggest ongoing gene flow between south-eastern Europe and the Caucasus, with both contributing to the Baltic population, which appeared only recently. The population from the Peloponnese Peninsula in southern Greece forms a common genetic cluster with samples from south-eastern Europe (Δ*K* approach in STRUCTURE, Principal Components Analysis [PCA]), although the results based on BAPS and the estimated likelihood in STRUCTURE indicate that Peloponnesian jackals may represent a distinct population. Moreover, analyses of population structure also suggest either genetic distinctiveness of the island population from Samos near the coast of Asia Minor (BAPS, most STRUCTURE, PCA), or possibly its connection with the Caucasus population (one analysis in STRUCTURE). We speculate from our results that ancient Mediterranean jackal populations have persisted to the present day, and have merged with jackals colonising from Asia. These data also suggest that new populations of the golden jackal may be founded by long-distance dispersal, and thus should not be treated as an invasive alien species, i.e. an organism that is “non-native to an ecosystem, and which may cause economic or environmental harm or adversely affect human health”. These insights into the genetic structure and ancestry of Baltic jackals have important implications for management and conservation of jackals in Europe. The golden jackal is listed as an Annex V species in the EU Habitats Directive and as such, considering also the results presented here, should be legally protected in all EU member states.

## Introduction

An implementation of molecular techniques to study population genetics has broadened our knowledge about several aspects of wildlife biology and ecology, including breeding characteristics [1, 2], population connectivity, and dispersal [3, 4]. Simultaneously, it enabled us to assess effects of historical processes [5–7], habitat fragmentation and isolation on distribution of genetic diversity (e.g. [8–10]) and to reconstruct routes of recent colonisations, range expansions and biological invasions [11–14]. As a result, information provided from molecular markers is frequently used in wildlife management and conservation of endangered species [15–19].

Changes in the geographical range are recognized as natural processes and have occurred in the history of most species [20–23]. Theoretical aspects of genetic after-effects of range shifts have been thoroughly analysed (e.g. [24]). It was shown that range expansions may lead to changes in population genetic structure and diversity. Initially, genetic structure should be clearly emphasized and genetic diversity in subdivided population will likely be reduced in comparison with the main distribution range and/or source population due to repeated bottlenecks. However, over time as new areas are occupied, connectivity among territories may be established and spatial population structure might decrease due to balanced gene flow among populations, causing homogenization and increased genetic diversity within populations [24–26]. Surprisingly, the genetic consequences of natural, contemporary range expansions have begun to be investigated only very recently [27–31] and results so far are equivocal and not always concordant with theoretical expectations.

Several carnivore species are currently expanding their distributions, especially in Europe [32]. It has been observed that such populations are characterized by particular genetic structure and processes, at least on the scale of individual countries. The study of the recently expanding (most probably from Russia) brown bear (*Ursus arctos*) population in Finland revealed disappearance of initial structuring and homogenization, as well as gradual increase of genetic diversity [33], as expected on the basis of theoretical models of range expansion [24, 25, 34]. Moreover, Hagen et al. [33] have shown increasing admixture between two genetic clusters occurring in Finland [35] as the range expansion proceeded. In contrast, the Finnish grey wolf (*Canis lupus*) population, also expanding since the 1990’s after almost complete eradication in the 19th century, exhibited decreased genetic diversity during the initial phase of expansion, despite clearly lower estimated population size [36]. The authors attributed this result to a low degree of connectivity with adjacent Russian wolf population.

The golden jackal (*Canis aureus*) is one of the most widely-distributed canid species, found in many areas of Europe and southern Asia [37, 38]. The ongoing expansion of the species in Europe has caused concerns in regard to possible negative effects its presence could exert, for example through excessive predation of other wildlife species or livestock, and the transmission of pathogens. In addition, there are several uncertainties regarding jackal management and policies, often in association with the unknown origins of jackal populations [38].

Population genetics of this species has been so far poorly characterised, especially when compared to Europe’s large carnivores, such as the grey wolf (e.g. [39–42]), the European lynx (*Lynx lynx*) [43–47] or the brown bear (e.g. [35, 48–50]). The first study focused on jackals in Serbia [51] suggested a low level of genetic diversity and weakly pronounced genetic structure in this recently-spreading population (see also [52]). Low genetic differentiation was also found in populations from Bulgaria, Croatia, and Italy [52]. A significant but weakly-pronounced genetic structure was only observed in the population of jackals from Dalmatia (Adriatic coast of Croatia). Fabbri et al. [52] also discovered that the jackals in Italy have an admixed origin from the Dalmatian and mainland populations. The genetic data in these cases were suggestive of a colonization process in golden jackals that is predominantly of a ‘stepping-stone’ nature, with short-distance dispersal and intermediate admixture. This contrasts with the long-distance dispersal observed in other canids, such as grey wolves [53, 54].

Genetic relationships of the European golden jackals with jackals from the Asiatic part of the species’ range, were not yet determined. Moreover, none of the studies so far analysed genetic structure of the population on the larger scale (i.e., the continental level). Consequently, the understanding of historic development of jackal populations in Europe is lacking. One of the hypotheses suggested that the European population goes back to the introduction of jackals from northern Africa in the 15th century [55]. This was later rejected on the basis of morphology [56, 57], but the origin of most of the European population remains unknown. Archaeologic data indicate that jackals were already present along the Mediterranean coast in Croatia and Greece ca. 7,000–6,500 yBP [58, 59]. Jackals remained absent from most of Europe until the 19th century, when the species started to expand slowly, followed by a rapid expansion at the end of the 20th century, which continues today [38, 60]. However, it is unclear whether any of the present European populations originate from this ancient Mediteranean population or if they are decendants of the later Asian colonization, e.g. from the Middle East or the Caucasus. Secondly, if there was a recent colonization from the east, it is unknown whether original small Mediterranean populations survived and merged with the wave of recent expansion. It is also unknown whether low genetic diversity and lack of distinct genetic structure in part of the European golden jackal population [51, 52] is an after-effect of fragmentation and population decline in the first half of 20th century, or rather resulted from recent expansion, interlinked with the founder effect pertaining to a recently established population. Hence, samples from potentially long-lasting, stable populations, such as southern Greece, should be analyzed. Although it was suggested that Italy was colonised from the Dalmatian coast and the mainland [52], the source of other expansions in Europe have not yet been identified. The lack of proper knowledge about the history of golden jackals in Europe can significantly affect management decisions and thus influence the conservation of the species. For example, the Estonian, Latvian and Lithuanian governments, despite the lack of reliable data, consider the golden jackal to be an alien species introduced to the Baltics by people, and based on this, these governments recently allowed unlimited lethal removal with the goal of eradicating the species [38].

The aim of the present work is to characterise for the first time the population genetic structure of European golden jackals on the continental scale, with the incorporation of samples from hitherto unstudied regions. Therefore, we included samples from the Peloponnesus Peninsula (southern Greece), which could possibly originate from the Neolithic population [59]; the insular population on the island of Samos located 1.7 km from the coast of Asia Minor, which represents the first investigation of an island population of the species; and the population from the Caucasus, a region known as a ‘hotspot’ for biodiversity [61]. An attempt is also made to identify the origin of the recently-established population in the Baltic States, and hence to resolve its controversial status and aid management decisions.

## Material and Methods

### Samples

**Fig 1:**
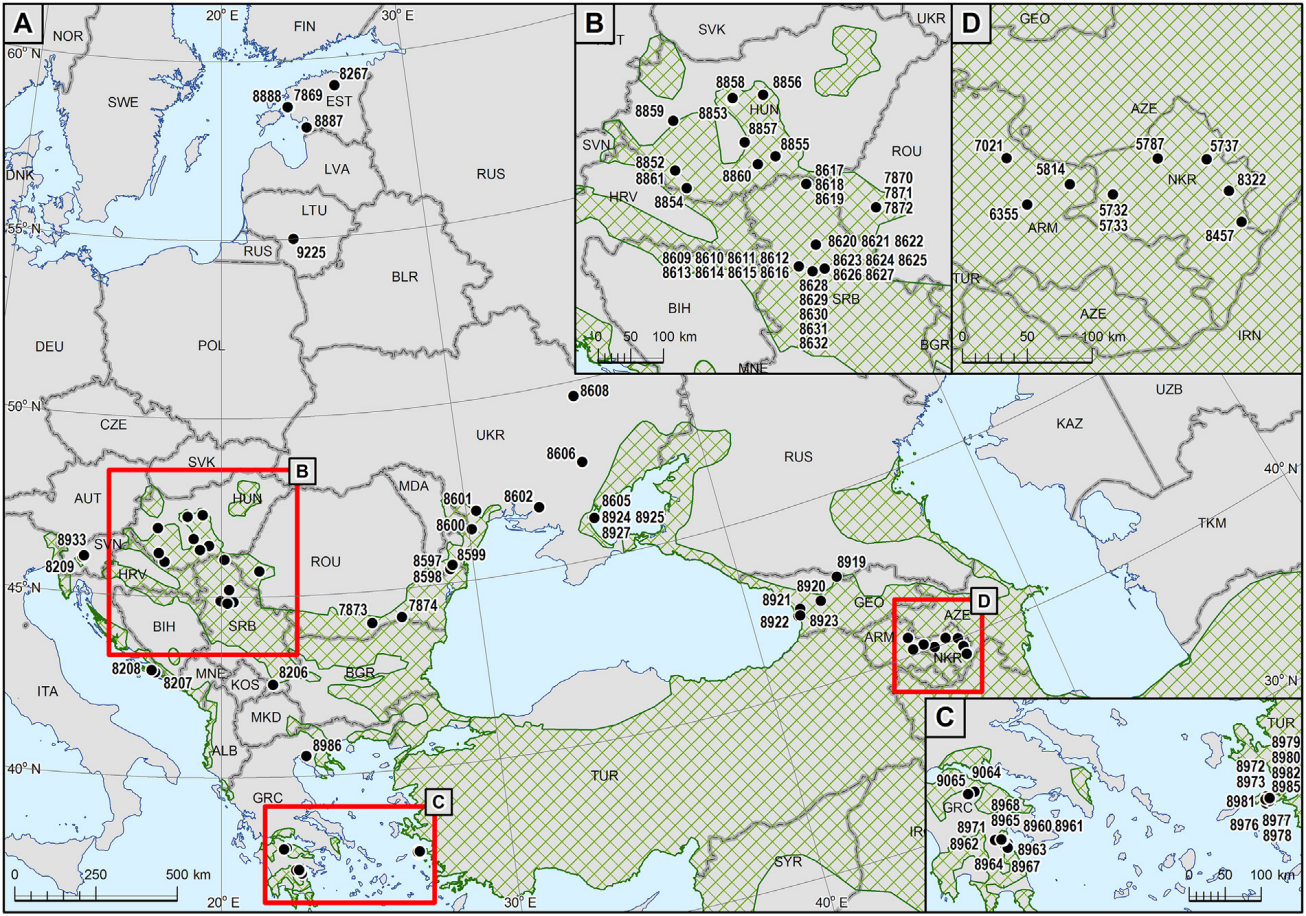
Distribution of sampling sites. Shaded areas represent areas with permanent presence of jackals (based on [38] and [37]).

Tissue samples used in this study were obtained from 97 individuals originating from five geographical regions (Fig 1, Table A in S1 File), i.e. i) south-eastern Europe (SEE)—comprising samples from Romania (country code ROU; *n* = 5), Croatia (HRV; *n* = 2), Slovenia (SVN; *n* = 2), Ukraine (UKR; *n* = 12), Serbia (SRB; *n* = 25), Hungary (HUN; *n* = 10), and northern Greece (GRC; Chalkidiki Peninsula, *n* = 1); ii) the Caucasus (CAU)—comprising samples from Mountainous Karabakh (NKR; *n* = 6), Armenia (ARM; *n* = 3), and Georgia (GEO; *n* = 5); iii) the Baltic States (BAL)—comprising samples from Lithuania (LTU; *n* = 1) and Estonia (EST; *n* = 4); iv) southern Greece (GRE-P) —comprising samples from the Peloponnese (GRC; *n* = 11); and v) the island of Samos (GRE-S, *n* = 10).

### Molecular genetics protocols

Genomic DNA was extracted using NucleoSpin Tissue kit (MACHEREY-NAGEL) with standard protocol. We amplified 15 microsatellite loci: CPH4, CPH5, CPH8, CPH12, CPH6, CPH9 [62], CPH22 [63], FH2004, FH2088, FH2096, FH2137, FH2140 [64], CXX.213, C09.250, C20.253 [65] (Table B in S1 File), as their polymorphism was shown in the golden jackal [52]. This number of polymorphic markers is efficient to detect genetic structure and describe genetic diversity within populations [66]. For 12 loci PCR were performed in 15 μl containing 1 μl of DNA, 1 μl of 8 μM primer mix, 7.5 μl of Multiplex PCR (Qiagen). Twelve loci were amplified in three multiplexing sets at following thermal profile: 95°C for 15 min, 40 cycles at 94°C for 30s, 57°C for 90 s, 72°C for 90s and final extension at 72°C for 10 min. The last three loci were amplified individually in total volume of 15 μl containing 1 μl of DNA, 0.5 μl of each 10 μM primers, 7.5 μl PCR Master Mix (EURx). The thermal profile was 95°C for 3 min, 35 cycles at 95°C for 30 s, 57°C for 45 s, 72°C for 45 s and final extension at 72°C for 5 min. PCR products were analyzed in a CEQ8000 sequencer (Beckman Coulter) and allele sizes were estimated using the Beckman Coulter Fragment Analysis Software.

Amplification of hypervariable domain of the mitochondrial DNA (mtDNA) control-region was performed with primers WDLOOPL (5’-TCCCTGACACCCCTACATTC-3’) and H576 (5-CGTTGCGGTCATAGGTGAG-3’) [52]. The PCR reaction mixture containing 2 μl of DNA, 1 μl of each 10 μM primers, 20 μl of PCR Master Mix (EURx) and 16 μl of purified water. The PCR profile was 94°C for 2 min, 40 cycles at 94°C for 15 s, 55°C for 20 s, 72°C for 60 s and final extension at 72°C for 2 min. Amplified products were purified using Clean-up kit (A&A Biotechnology), and then sequenced using BigDye Terminator v3.1 Cycle Sequencing Kit and 3500xL Genetic Analyzer (Applied Biosystems).

### Statistical analysis—microsatellites

Polymorphism among microsatellite loci was estimated on three levels. Firstly, we estimated the number of alleles (*A*), observed heterozygosity (*H*_O_), unbiased expected heterozygosity (*H*_E_, [67]) and inbreeding coefficient (*F*_IS_) for each locus in the total sample (*N* = 96). The significance of *F*_IS_ was tested under a randomization procedure, with the Bonferroni correction for multiple comparison. These analyses were performed using GenAlEx version 6.5 [68] and FSTAT version 2.9.3.2 [69]. In addition, a probability test for deviation from the Hardy-Weinberg equilibrium (HWE) was evaluated for each locus using Genepop (Web version 4.2; [70, 71]). Secondly, we estimated polymorphism for each locus in groups of samples designated *a priori* and corresponding with geographical regions. Aside from *A*, *H*_O_, *H*_E_ and *F*_IS_, we also calculated allelic richness (*R*; [72]) using FSTAT, as well as mean values for these parameters. HWE was tested for each locus within each region, as well as for each region across all loci. Between-populations genetic differentiation was estimated using *F*_ST_ [73] as implemented in FSTAT.

**Fig 2:**
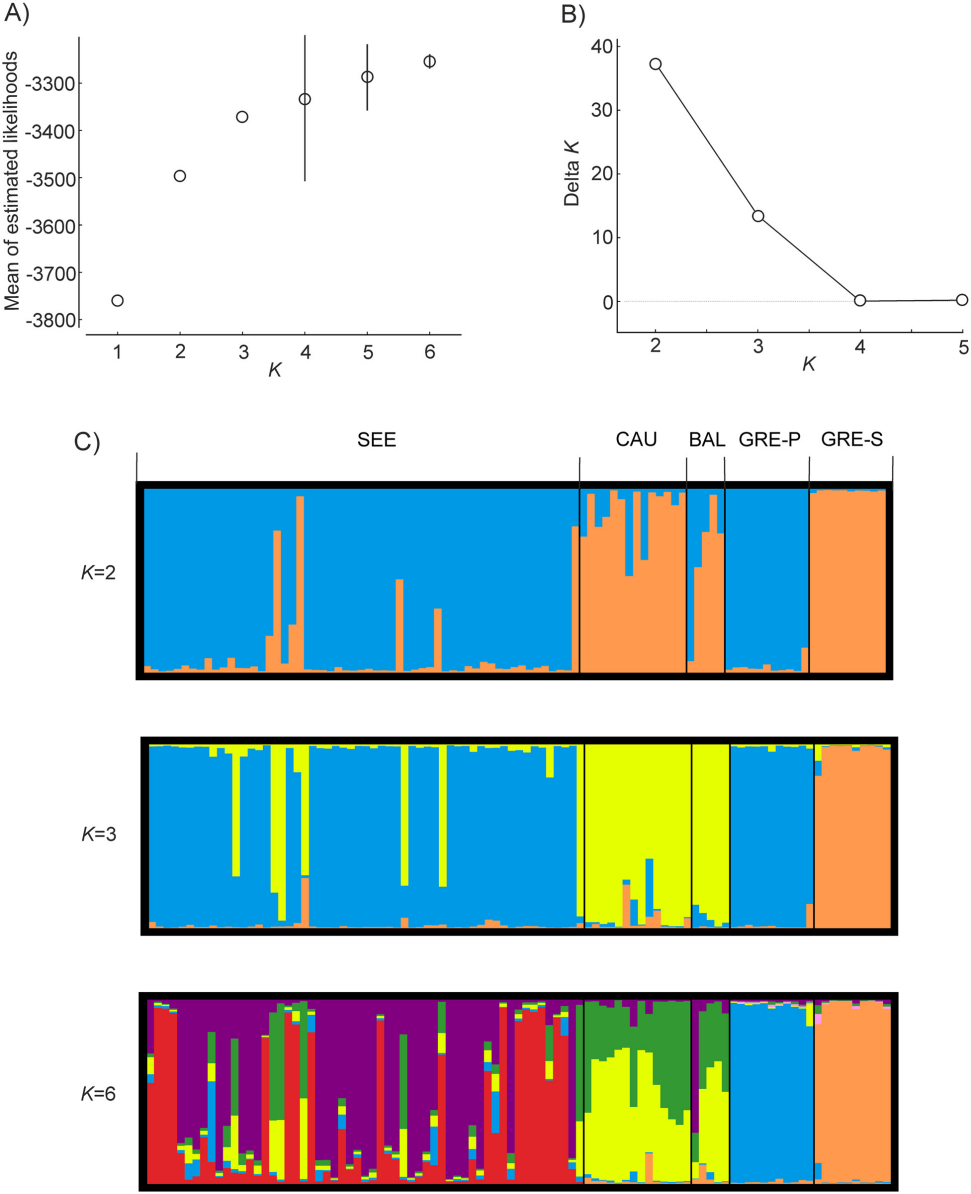
STRUCTURE results: A—estimated likelihoods, ln P(D), of each number of inferred genetic clusters (bars are SD—only given when exceeding the width of dots); B—the corresponding Δ*K* curves as a function of *K*; C—ancestry of individuals, estimated for *K* = 2 and 3 (based on Δ*K*), and 6 (based on estimated likelihoods). SEE—south-eastern Europe; CAU—Caucasus; BAL—Baltics; GRE-P—Greece, Peloponnese; GRE-S—Greece, Samos Island.

To find out whether the geographical grouping of samples corresponded with genetic groups, we applied a Bayesian-clustering method (STRUCTURE version 2.3.4; [74]). Structure was run 15 times for each user-defined number of genetic groups (*K* = 1–6), with an initial burn-in of 50,000, and 1,000,000 iterations of the total data set. The admixture model of ancestry and the correlated model of allele frequencies were applied. Sampling location was not used as prior information. Next, we examined Δ*K* statistics that identify the largest change in the estimates of *K* produced by STRUCTURE (Fig 2A versus Fig 2B) [75]. To visualise the STRUCTURE results we used STRUCTURE HARVESTER 0.6.94 [76]. We then applied CLUMPP 1.1.2 [77] to average the multiple runs given by STRUCTURE and correct for the label switching. The output from CLUMPP was visualised using DISTRUCT v 1.1 [78].

The Bayesian-based method implemented in the Bayesian Analysis of Population Structure software (BAPS, version 6.0; [79–81]) was used to check the spatial clustering of individuals, and was followed by admixture analysis. In this analysis, geographical coordinates for each sample were used. Ten replicates were run for every upper level of *K* (2, 3, 4, 5, 10, 15, and 20). The number of iterations used to estimate the admixture coefficient for individuals, and the number of reference individuals from each location were 50 and 200, respectively. The number of iterations applied to estimate the admixture for reference individuals was set at 15.

We also obtained an additional representation of the genetic structure using Principal Components Analysis (PCA). This multivariate descriptive method is not dependent on any model assumption and can thus provide a useful validation of the Bayesian clustering output [82–84]. We used the R package ADEGENET v1.3.4 [85] to carry out the standard PCA. The results of the analysis were presented graphically along first and second axes in line with eigenvalues.

### Statistical analysis—mitochondrial DNA

Sequences were aligned in BioEdit software v.7.0.5.3 [86], with alignments then being checked manually. We amplified a 406 base-pair (bp) fragment of the control region for 93 samples also genotyped with microsatellite markers. We were unable to obtain reliable sequences from one sample from Estonia (EST), one from the Caucasus (CAU), and two from south-eastern Europe (SEE). Numbers of haplotypes (*H*) in the total sample, as well as in particular geographical regions and genetic groups, haplotype diversity (*h*), nucleotide diversity (π) and mean number of nucleotide differences among haplotypes (*k*) were all calculated using DNAsp 5.10 [87]. Haplotype frequencies in the overall sample and in each geographical region were calculated using ARLEQUIN v3.5.1.2 [88]. ARLEQUIN was also used to calculate pairwise *θ*_ST_ among regions using haplotype frequencies. The test for significance was performed with 1,000 permutations. The overall genetic structure, based on haplotype frequencies, was estimated in DNAsp, using *H*_ST_ ([89]; equation 2). Significance for the global estimate was determined by permutation test, on the basis of 1,000 replicates.

A median-joining haplotype network [90] was constructed in NETWORK v4.6.1.1. (Fluxus Technology Ltd.). We also compared haplotypes identified in this study (GenBank accession nos. KT362174–KT362176) with haplotypes for the golden jackal deposited in GenBank, and originating from Bulgaria, Serbia, Croatia, and Italy (KF588364) [51, 52], Serbia (HQ845260) [91], Bulgaria (AF184048) [92], Poland and Ukraine (KT268318 and KT268319) [93], the Caucasus (KJ490945 and KJ490946) [94], and India (AY289997 and AY289996) [95].

### Ethics Statement

Tissue samples used in this study were obtained from individuals that died in vehicle collisions, due to natural causes or as a result of legal hunting. No animal was killed for the purpose of this study.

## Results

### Microsatellites

From 15 polymorphic microsatellite loci, amplified in 97 golden jackals (Fig 1), we identified 102 alleles (1.05 alleles per individual). At most loci the polymorphism was moderate (5 to 11 alleles). The greatest number of alleles (*A* = 14) was discovered at locus FH2137, the lowest (*A* = 3) at CPH5 (Table C in S1 File). In most cases the observed heterozygosity was below 0.60, and at only three loci (FH2004, FH2096, FH2137) did the value exceed 0.70. When all samples were analysed together, 11 of the 15 microsatellites were found not to be in HWE (Table C in S1 File). Similarly, *F*_IS_ values were found to differ significantly from zero at most loci following Bonferroni correction, the effect being indicative of heterozygote deficiency. Given that all the samples were examined together, and we subsequently found significant substructure, this could be due to the Wahlund effect.

When samples were grouped by geographical distribution, a significant overall *F*_IS_ was found only in the case of jackals from the Caucasus (CAU). In south-eastern Europe (SEE)—the region represented by the highest number of the samples studied—there were three loci manifesting deviation from the HWE on account of heterozygote deficiency and one, FH2096, indicative of heterozygote excess (Table D in S1 File). SEE was also the only group with significant overall heterozygote deficiency, though *F*_IS_ was low and non-significant. This group also had the highest mean number of alleles (mean *A* = 5.40). Allelic richness was similar in SEE, CAU and BAL, though slightly lower in two groups from southern Greece, i.e. from the Peloponnese (GRE-P) and Samos (GRE-S). Observed heterozygosity (*H*_O_) was highest in SEE. The lowest *H*_O_ was found in the insular GRE-S population.

Analysis of genetic structure using Bayesian methods and PCA indicated some grouping patterns. In the STRUCTURE analysis the highest mean likelihood was indicated for six clusters (Fig 2A). GRE-P and GRE-S formed two uniform genetic groups, whereas SEE consisted mainly of individuals from two clusters (with most jackals from Hungary and Romania marked in red, and the majority of those from Serbia and Ukraine shown in violet—Fig 2C; *K* = 6), but also of individuals of mixed ancestry. Jackals from CAU and BAL were assigned to two other clusters, with more or less equal probability of ancestry from each of them. The Δ*K* statistic (Fig 2B) suggested two or three genetic groups. In the two-group scenario the first cluster comprised the majority of individuals from SEE and GRE-P, and the second comprised the majority of individuals from GRE-S, CAU and BAL (Fig 2C; *K* = 2). On the basis of the *K* = 3 value, BAL and CAU formed the first genetic group, SEE and GRE-P the second, and GRE-S the third (Fig 2C; *K* = 3). In both of these cases, certain individuals from SEE had the highest probability of ancestry from the CAU/BAL group. These were four individuals from Ukraine (nos. 8599, 8607, 8608, 8927) and two individuals from Serbia (nos. 8620, 8625—Fig 1).

**Fig 3:**
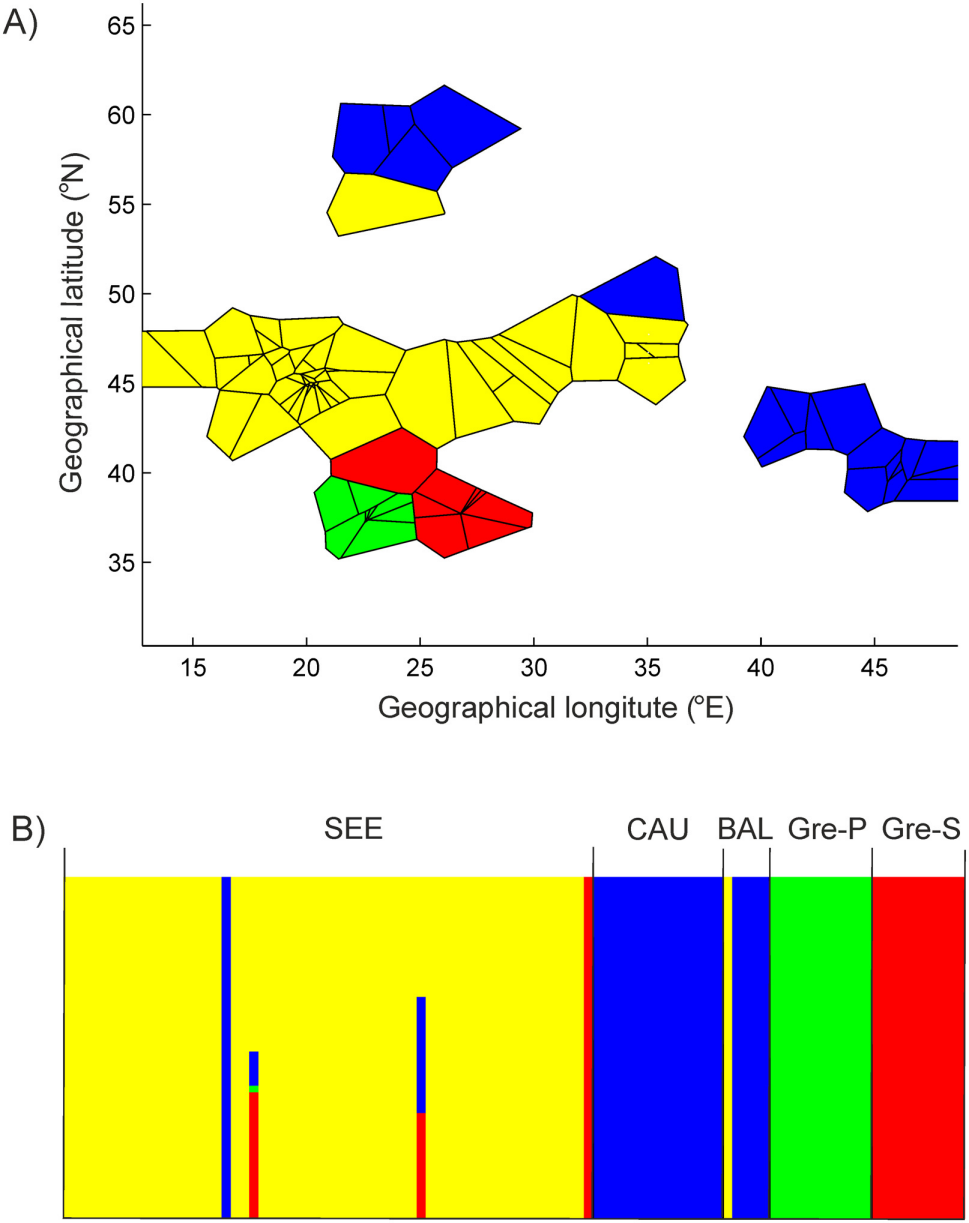
Results of spatial analysis of genetic structure, using BAPS: A—assignment of specimens to four genetic clusters indicated by spatial clustering; B—admixture analysis of identified clusters. SEE—south-eastern Europe; CAU—Caucasus; BAL—Baltics; GRE-P—Greece, Peloponnese; GRE-S—Greece, Samos Island.

**Fig 4:**
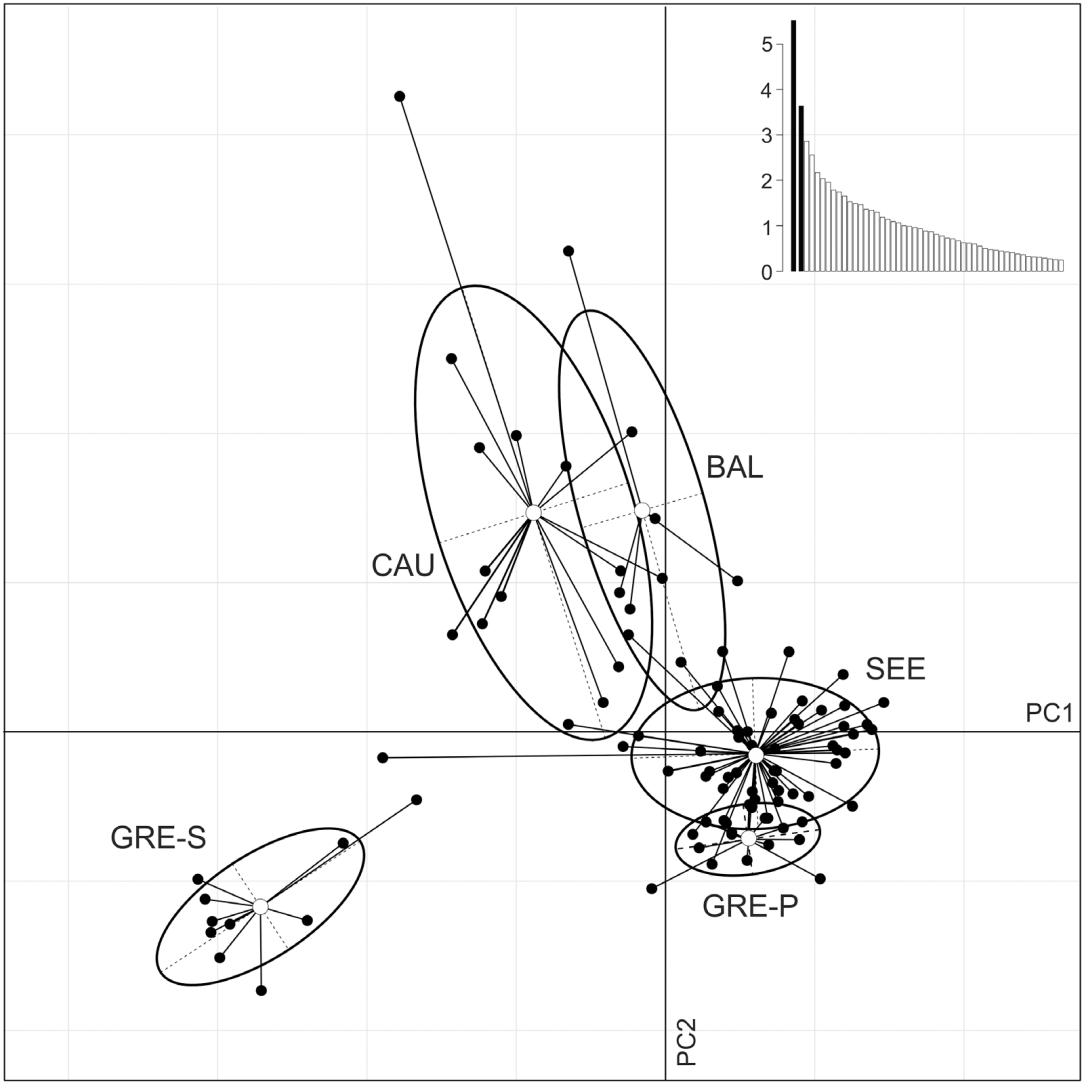
Results of Principal Components Analysis performed in ADEGENET. First and second axes and corresponding eigenvalues (inset) are shown. SEE—south-eastern Europe; CAU—Caucasus; BAL—Baltics; GRE-P—Greece, Peloponnese; GRE-S—Greece, Samos Island.

Geographical information about samples in Bayesian analysis (BAPS) suggested the presence of four genetic groups, with a very limited admixture among them (Fig 3A and 3B). In general, the geographical groups designated *a priori* corresponded to genetic groups as indicated by BAPS. However, one sample from SEE (Ukraine, no. 8608) was assigned to the CAU/BAL cluster, one sample from SEE (northern Greece, no. 8986) was assigned to the GRE-S cluster, and one sample from BAL (Lithuania, no. 9225) was assigned to SEE (Fig 1). A similar result was obtained by way of admixture analysis (Fig 3B), although in this case two additional individuals from SEE (nos. 8927 and 8625 from Ukraine and Serbia, respectively) were found to be of mixed ancestry. Like STRUCTURE, PCA pointed to the genetic distinctness of GRE-S (Fig 4). The remaining samples were divided by PCA into two groups corresponding with SEE/GRE-P and CAU/BAL.

Genetic differentiation among the geographical regions was high (overall *F*_ST_ = 0.199, 95% CI = 0.147–0.258). Pairwise *F*_ST_ ranged from 0.05 to 0.39 (Table 1). Low genetic differentiation was found between BAL and CAU, whereas all pairwise comparisons with GRE-S indicated a very high level of genetic differentiation (*F*_ST_ > 0.20). Similarly, marked genetic differentiation was found between GRE-P and CAU and GRE-P and BAL, while moderate genetic differentiation characterised the pairwise comparisons of data for SEE, as set against GRE-P, BAL or CAU.

**Table 1:** Genetic differentiation among geographical regions: SEE—south-eastern Europe (Croatia, Serbia, Slovenia, Hungary, Romania, Ukraine, northern Greece); CAU—Caucasus (Georgia, Armenia, Mountainous Karabakh); BAL—Baltics (Estonia, Lithuania); GRE-P—Greece, Peloponnese; GRE-S—Greece, Samos Island. Above diagonal—genetic differentiation calculated from mtDNA haplotype frequencies, below diagonal—genetic differentiation calculated from microsatellites. Significant values (1,000 permutations; *P* < 0.05) are shown in bold.

Region	SEE	CAU	BAL	GRE-P	GRE-S
SEE		**0.347**	-0.199	**0.507**	**0.961**
CAU	**0.125**		-0.045	0.024	**0.716**
BAL	**0.100**	0.051		0.090	**0.863**
GRE-P	**0.113**	**0.207**	**0.268**		**0.790**
GRE-S	**0.293**	**0.205**	**0.343**	**0.388**	

### Mitochondrial DNA

Based on the mitochondrial DNA (mtDNA) control region fragment, we identified four unique haplotypes in 93 samples. Both haplotype diversity and nucleotide diversity were low (Table 2), as was the average number of pairwise nucleotide differences (*k* = 0.706). Apart from BAL, we identified two haplotypes per region. The highest level of haplotype diversity was found in GRE-P, while the most marked nucleotide diversity and highest average number of pairwise nucleotide differences was found in CAU (Table 2).

**Table 2:** Sample size (*N*) and genetic characteristics of mtDNA polymorphism in *C*. *aureus* in geographical regions and all samples: *h*—number of identified haplotypes; *H* [SD]—haplotype diversity and corresponding standard deviation; π [SD]—nucleotide diversity and corresponding standard deviation; *k*—average number of pairwise nucleotide differences. SEE—south-eastern Europe; CAU—Caucasus; BAL—Baltics; GRE-P—Greece, Peloponnese; GRE-S—Greece, Samos Island.

Region	*N*	*h*	*H* [SD]	π [SD]	*k*
SEE	55	2	0.036 [0.035]	0.00009 [0.00009]	0.036
CAU	13	2	0.385 [0.132]	0.00189 [0.00065]	0.769
BAL	4	1	-	-	-
GRE-P	11	2	0.509 [0.101]	0.00125 [0.00025]	0.509
GRE-S	10	2	0.467 [0.132]	0.00115 [0.00032]	0.467
Total	93	4	0.344 [0.061]	0.0017 [0.00033]	0.706

**Fig 5:**
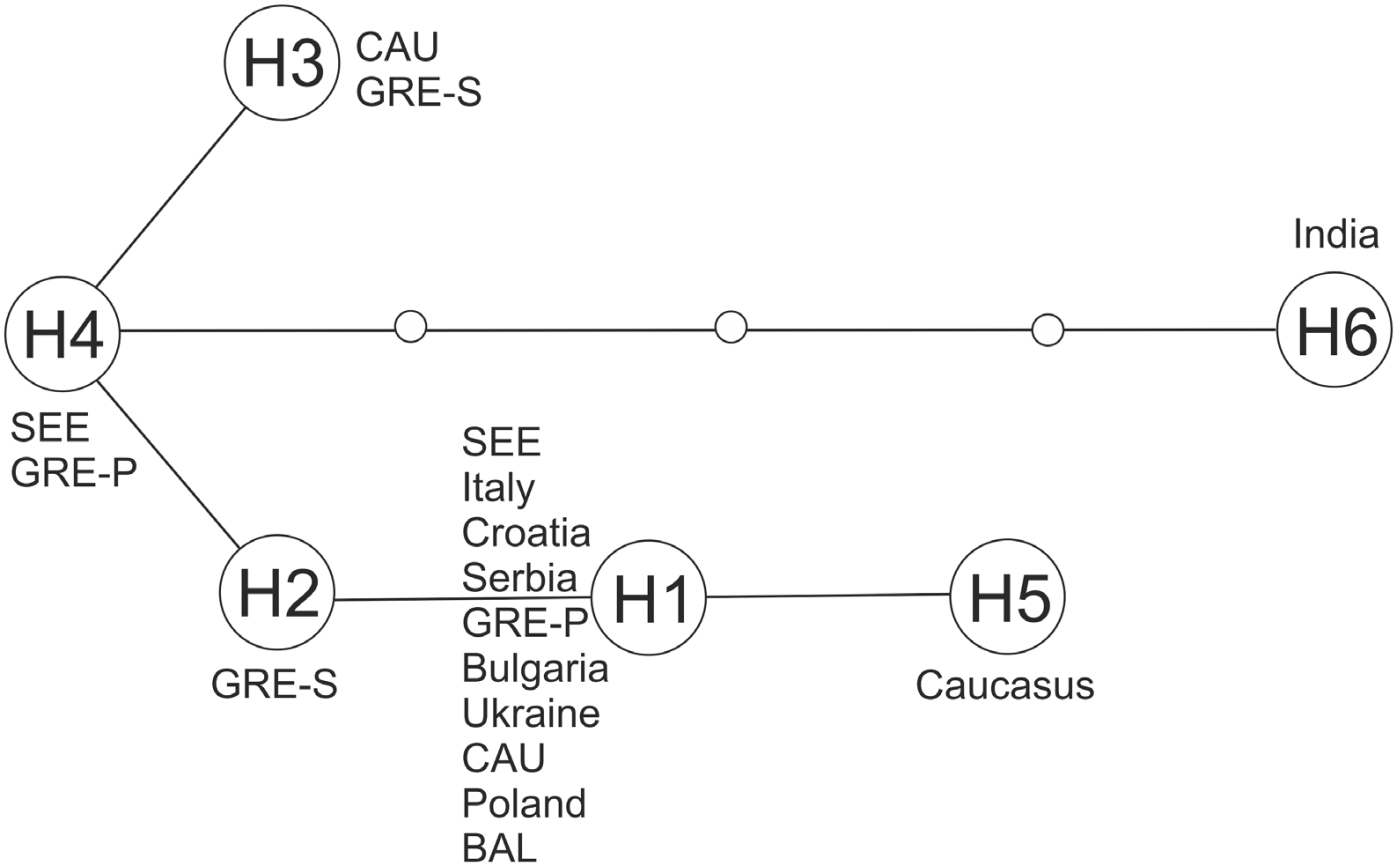
The minimum spanning network of mtDNA haplotypes of golden jackals sampled in this study (SEE, CAU, BAL, GRE-P, GRE-S) as well as those desposited in GenBank (Italy, Croatia, Serbia, Bulgaria, Ukraine, NW Poland, the Caucasus, and India). The length of each line between two circles is proportional to the number of mutations.

Haplotype H1 proved to be most frequent, being absent only from GRE-S. In BAL this was the only haplotype found. Haplotype H2 proved to be unique to GRE-S, while H3 was shared between CAU and GRE-S, and H4 between SEE and GRE-P (Table 3, Fig 5).

**Table 3:** Distribution of golden jackal mtDNA haplotypes in the investigated geographical regions. Frequency in the region and overall frequencies are reported. SEE—south-eastern Europe; CAU—Caucasus; BAL—Baltics; GRE-P—Greece, Peloponnese; GRE-S—Greece, Samos Island.

Haplotype	Motif	SEE	GRE-P	CAU	BAL	GRE-S	Total
H1	TGG	0.98	0.64	0.77	1.00	-	0.800
H2	CAA	-	-	-	-	0.70	0.076
H3	TAA	-	-	0.23	-	0.30	0.068
H4	TAG	0.02	0.36	-	-	-	0.056

Comparing haplotypes identified in this study with those deposited in GenBank (homological sequences of the 250 bp of mtDNA CR), we found that H1 corresponds with a haplotype identified previously in Italy, Croatia, Serbia, Bulgaria, Ukraine, NW Poland, and the Caucasus, while differing by just a single mutation from another haplotype from the Caucasus (H5). A haplotype observed previously in Indian jackals (H6) differed from H4 by just four mutations (Fig 5).

Genetic structure as estimated on the basis of haplotype frequencies was found to be pronounced and significant (*H*_ST_ = 0.486 for geographical groups, *P* < 0.001). Pairwise *θ*_ST_ was highest for the comparison of SEE with GRE-S. No genetic differentiation was noted between BAL and CAU or between BAL and SEE (Table 1).

## Discussion

### Genetic diversity

Analysing the results obtained with both microsatellite and mitochondrial markers, we found higher genetic diversity than has been reported previously for other European populations of the golden jackal [51, 52], except in the case of the island population from Samos. In Serbia [51] a total of 31 microsatellite alleles at eight loci were found in 120 individuals, giving 3.8 alleles per locus and 0.26 alleles per individual, compared with 6.8 alleles per locus and 1.05 alleles per individual stated in our study (Table C in S1 File). Similarly, in the Serbian population the total observed heterozygosity was 0.28, compared with the 0.52 found in our study. These differences can be explained by the fact that the populations of golden jackals analysed in this study were historically older and larger than those from Serbia, or involved samples from across a larger area, with the SEE geographical group encompassing individuals from a large part of south-eastern, Central and Eastern Europe. Furthermore, the mean number of alleles was higher in SEE and CAU (*A* = 5.40 and 4.67, respectively; Table C in S1 File) than that found previously [52] in the contemporary samples from Bulgaria (*A* = 3.5), Slavonia (continental eastern part of Croatia) and Serbia (*A* = 4.0), Dalmatia (*A* = 2.8), and Italy (*A* = 3.7). Moreover, the analysis of the mitochondrial control region revealed four mitochondrial haplotypes (Table 3), as opposed to the one haplotype noted in previous studies [51, 52]. However, the population from Greece (both the Peloponnese and the island of Samos), had a mean number of alleles of around 3.0 (Table C in S1 File), comparable with what was found in Dalmatia [52], and hence slightly lower than the value characterising jackals in Slavonia and Serbia, Bulgaria and Italy [52]. We observed the lowest level of genetic diversity in the island population at Samos (mean *A* = 2.67, *H*_O_ = 0.38; Table C in S1 File), which could be explained by the isolation, as low genetic diversity often reflects colonisation of an island by a small number of individuals (the founder effect) and random processes reducing variability, such as genetic drift [96–98].

In the present continent-wide study we supported previous findings of Zachos et al. [51] and Fabbri et al. [52] in Serbia, Bulgaria, Croatia, and Italy, indicating that Europe’s golden jackals harbor less genetic diversity compared to other wild canids, such as wolves [36, 39–41, 99, 100], or red foxes (*Vulpes vulpes*) [101, 102]. The genetic diversity of European jackals is also clearly lower than that found in jackals from Israel [103], which show signals of hybridization with grey wolves, dogs, and the African golden wolf (*Canis anthus*) [104]. For example, the mean number of alleles in five populations from Israel ranged from 4.7 to 5.6 versus 2.6–5.4 noted in our study, whereas observed heterozygosity ranged from 0.64 to 0.72 versus 0.38–0.55 in our study. This is despite the dramatic population decline and bottleneck experienced in Israel in the 1960s [55, 105]. Thus the low genetic diversity of Europe’s jackals does not reflect species-specific characteristics, but may be related to the unique history of golden jackals on this continent.

In contrast with the authors of previous studies [51, 52], we noted polymorphisms in the mtDNA control region, even though overall haplotype diversity was low (*H* = 0.34) with just four haplotypes despite the large sampling area. In jackals from the mainland sites (SEE, CAU, BAL, GRE-P), it was the haplotype recorded in previous studies (H1) that was found to occur most frequently. However, it was absent from the island population (Samos), where the unique H2 haplotype is prevalent. Higher mtDNA diversity compared with previous studies is mainly connected with the larger sampling area including the Caucasus (one ‘new’ haplotype H3) and Greece (three ‘new’ haplotypes: H2, H3 and H4). The highest level of haplotype diversity was found in the Peloponnese. The greater number of haplotypes in the Aegean region could suggest that the present population in Greece may, at least partially, descend from the ancient Greek population. However, confirmation of this hypothesis requires further study, preferably including fossil material. Moreover, in the south-eastern European population, alongside the haplotype discovered by Zachos et al. [51] and Fabbri et al. [52], we identified the additional haplotype H4, which appeared in one animal from the Biruchiy Peninsula (southern Ukraine), i.e., an area outside the Balkan Peninsula, but also in the Peloponnese. Hence, it is possible that the majority of the Balkan population of *C*. *aureus* is uniform in regard to control-region polymorphisms, as suggested by earlier studies [51, 52]. Hence, despite the discovery of additional haplotypes, the genetic diversity in the mitochondrial control region in Europe’s golden jackals should be regarded as low when compared with that in other canids [102, 106, 107]. However, further sampling will probably result in the detection of new polymorphisms in mtDNA of the golden jackal as a species, as the haplotype found in Indian jackals (denoted as H6 in Fig 5) differs by 4–6 substitutions from the haplotypes identified in the present study.

### Genetic structure

Previous studies of golden jackals in Europe emphasized the limited degree of genetic structuring, with only the coastal population from Dalmatia clearly differentiated from other Balkan samples [51, 52]. A genetic identity relating to Dalmatia has also been suggested in the case of the grey wolf [108], and was explained either by reference to an origin of this population in a distinct refugium, or in terms of ecological and behavioural factors [41, 109, 110]. Fabbri et al. [52] also noted markedly smaller number of alleles (*A* = 2.8) and more limited heterozygosity (*H*_O_ = 0.37) in Dalmatian jackals and suggested a long-term isolation of this population. In respect to this, we also call to attention that golden jackals were present in southern Dalmatia already in the Middle Ages [111] and possibly even much earlier [58].

Our analysis extending to the whole of Europe has pointed to the existence of a pronounced genetic structure in relation to both nuclear and mitochondrial markers. Individuals from an extensive area of south-eastern Europe generally form a uniform genetic group, as already noted by Zachos et al. [51]. Fabbri et al. [52] also reported small genetic differentiation in microsatellite markers among populations from Bulgaria, Slavonia and Serbia. This probably reflects recent expansion of the species in this region. However, Greek samples indicate the existence of a distinct population in the Peloponnese (STRUCTURE, BAPS) (see also [112]), even if both haplotypes found in south-eastern Europe were also present in animals from this peninsula. We can speculate that our results support the hypothesis that an ancient Greek population survived in the Peloponnese to the present day, recently merging with a population expanding in from the east. A similar interpretation can be put forward in regard to Dalmatian jackals, as already suggested by Fabbri et al. [52]. Thus the two known areas with the early Holocene findings of jackals [58, 59] are also the only two areas in south-eastern Europe today that show higher genetic differentiation, giving further support for the continuous presence of ancient populations along the Mediterranean coast.

STRUCTURE and BAPS suggested ongoing gene flow between the Caucasus and Europe as well—some individuals from SEE had the highest probability of ancestry from the CAU/BAL cluster. Interestingly, when the microsatellite genotypes are concerned, an individual from south-eastern Europe (no. 8927; Table A in S1 File) with the additional haplotype H4 (which is frequently found in the Peloponnesus Peninsula), was identified as having ancestry from the Caucasus (STRUCTURE: two-clade and three-clade scenarios) or mixed ancestry from the Caucasus and Samos Island (BAPS: admixture analysis).

The island population of golden jackals on Samos was highly differentiated from those from other sampling sites (*F*_ST_, STRUCTURE: three-clade scenario, PCA, BAPS). Unfortunately, there was no access to samples from the Turkish mainland, so it remains unclear whether the geographical barrier of water restrics gene flow between the island and that mainland. However, our genetic data indicate that there are or were some connections between Samos and northern Greece (e.g. an individual no. 8986 sampled in Chalkidiki Peninsula [Fig 1, Table A in S1 File] was assigned to the Samos cluster).

### Stepping stone model or long-distance colonizers—on the origin of the Baltic jackals

First possible observations of jackals in the Baltics are known from 2011, when groups of several jackals were noted in Estonia [113]. In 2013 and 2014 several animals were shot, photographed, or detected during howling surveys in Estonia and Latvia [113, 114], and in 2015 the first jackal was shot in Lithuania [115]. Although several carnivore experts suggested that natural expansion was likely, the governments of the Baltic States decided to assume that jackals were introduced by humans [38].

The genetic data suggest that jackals from the Baltics originate from the Caucasus region (Estonian samples), and from the population expanding out of south-eastern Europe (Lithuanian case). This dual origin does not support the idea that jackals were introduced by humans, as it is unlikely that someone would capture jackals in different regions and smuggle them to the Baltics. Additionally, recent records of jackal occurrence from Slovakia, Ukraine, Belarus, and north-western and eastern Poland [38, 93, 113–116], suggest that both Caucasian and southeastern European populations are spreading towards the north. The presence of the Caucasian gene pool was also detected in animals from NE Ukraine, further supporting the hypothesis of natural expansion from the Caucasian region through Ukraine towards Estonia.

The dynamics of species’ range expansions depend on habitat connectivity, but also on dispersal ability [118] and habitat plasticity [119]. Two basic models were suggested for a dispersal through fragmented environment, where suitable habitat is distributed as a series of patches. In the ‘island model’ all patches are equally accessible, while in the stepping-stone model exchanges of individuals are restricted to adjacent populations [120]. Although previous genetic data suggested a ‘stepping-stone’ nature of golden jackal dispersal [52], our results indicate the possibility of long-distance dispersal in this species. This can also be supported by a review of literature data, which includes several records of sudden appearances of jackals far from other known populations. Such example include the (re)colonization of Hungary in the 19th century [60] with the closest known populations at that time being in Dalmatia, Croatia (at ca. a 300 km straight-line distance) or Bulgaria (400 km away).

Another case resembling the sudden occurrence of several jackals in the Baltics, refers to the first colonization of Slovenia in the mid-20th century. In winter 1952/3 several jackals suddenly appeared in Central Slovenia near Ljubljana, with reported observations of groups of up to six animals [121] and later shooting of two animals near Ljubljana and one at the foothills of the Julian Alps in NW Slovenia [122]. At that time, the closest jackal population was known from Ravni Kotari in Dalmatia, Croatia [123], approximately 210 km from Ljubljana. In this probable case of long-distance dispersal, jackals seem to have dispersed in a group, as it would be highly unlikely that several animals would appear independently at the same time in the same place so far from the closest population.

More recent records that can be considered potential cases of long-distance dispersal of jackals include:

- a male observed several times from 1996 and then shot in 1998 in Südbrandenburg in Germany [124] and an individual photographed four times in 2012 in the Bavarian Forest [125]. These records were 430 km and 270 km distant, respectively, from the closest-known reproducing population in eastern Austria and western Hungary;
- five photo-records of a jackal in 2011 in the Northwestern Alps of Switzerland [126], with the closest known reproducing population in NE Italy 450 km away;
- an individual shot in 2014 near Olevsk in Northern Ukraine [127], 430 km from the closest known population in Southern Ukraine and Moldova;
- an individual shot in 2012 near Tomašovka in Belarus [117], 410 km from the closest known reproducing population in Hungary;
- a young male found dead in April 2015 on a road in NW Poland, close to the German border [98], ca. 610 km from the reproducing population in NW Hungary;
- GPS-GSM collared 1.5 year old female, which travelled 220 km during 12 days in Hungary in 2014 (J. Lanszki unpubl. data).

Based on this review of jackal occurrences and our genetic data, we suggest that it is not uncommon for golden jackals to disperse over several hundred kilometers in human-dominated landscapes. This could explain the speed of jackal expansion in Europe that has been observed in the last decades [38]. We also suggest that the recent colonization of the Baltic States is most likely a case of long-distance dispersal. The first ‘wave’ of colonization of the Baltics appears to have originated from the Caucasus region via Ukraine. The second wave on the other hand seems to have originated from south-eastern Europe through an expansion front in Romania, Hungary/western Ukraine, Slovakia, and Poland. According to available records it even appears that a group of several jackals can disperse together (see also [116]). If true, this would have important implications, as it would considerably increase probability of successful colonization of new areas.

### Management and conservation implications

The golden jackal has already been declared an alien, potentially invasive species in all Baltic States (e.g. [128]). However, an Invasive Alien Species (IAS) needs to meet at least three criteria: 1) it should be non-native, allochtonous, introduced by people; 2) it should threaten biological diversity on the local scale; and 3) it should be characterised by rapid population growth [129]. Although exponential increase in population size has been observed (e.g. in Hungary [130]), the other two criteria have not been met. The movement north is evidently a result of natural migration (as the present study shows), and there is no proof of a harmful effect on local fauna [131–134]. Also there are no major complaints about golden jackals inflicting harm on domestic animals reported from Europe [133–136]. Occasional reported claims of jackal depredation of livestock are believed to be exaggerated often [134, 137], or connected with erroneous identification, when reported cases have been inspected using forensic genetics [138]. Recent genetic analysis [104] has also shown that the severe impacts on livestock reported from Israel [139], are probably not connected with golden jackals *per se*, but rather with individuals of admixed origin between several canid species. Furthemore, the parasite load in the European golden jackal is similar to or lower than that in other carnivores (e.g. the red fox, grey wolf, and wild cat [*Felis silvestris*]) in the region [140–143], and no attacks by jackals on people are known. For these various reasons, concerns regarding serious negative impacts of the expansion of the golden jackal in Europe appear to be unfounded as yet.

Nevertheless, results presented here have several management and conservation implications. The existence of long-distance dispersal in the golden jackal would seem to warrant the initiation of international coordination in management of the species in Europe and more focus on management at the population, rather than at the national level, especially considering considerable differences that currently exist among countries [38]. We therefore suggest the development of trans-boundary management strategies and documents similar to the population-level management approaches developed in the case of Europe’s large carnivores [144]. We also call for a revision of the approach used in managing jackals in the Baltic States, given that our results contradict the presumption of the local decision-makers about the human-assisted origin of the Baltic population. Lastly, our results provide a basis for the development of a conservation strategy for the golden jackal in the region. We propose that priority should be given to the Caucasus region, which harbors high genetic diversity in terms of the number of microsatellite alleles, as well as to the regions of the Peloponnese and Dalmatia [52], in which a relict gene pool from ancient Mediterranean populations appears to have persisted. The golden jackal is listed as an Annex V species in the EU Habitats Directive and as such, taking above into account, should be legally protected in all EU member states (for legal implications of range expansion in this species see [38]).

## Supporting Information

S1 File Material studied and its genetic characteristics. **Table A,** List of examined specimens including specimen number, sex, locality information, date, geographical coordinates, and mtDNA haplotype designation. **Table B,** Microsatellite genotypes. **Table C,** *Per locus* genetic diversity in 97 samples of golden jackal. **Table D,** *Per region* genetic diversity estimated based on polymorphisms in 15 microsatellite loci.
(DOC)

## References

[1] Avise JC, Jones AG, Walker D, DeWoody JA. Genetic mating systems and reproductive natural histories of fishes: lessons for ecology and evolution. Annu Rev Genet. 2002;36: 19–45. 12429685 10.1146/annurev.genet.36.030602.090831

[2] Griffith SC, Owens IPF, Thuman KA. Extra pair paternity in birds: A review of interspecific variation and adaptive function. Mol Ecol. 2002;11: 2195–2212. 12406233 10.1046/j.1365-294x.2002.01613.x

[3] Broquet T, Petit EJ. Molecular estimation of dispersal for ecology and population genetics. Annu Rev Ecol Evol Syst. 2009;40: 193–216. 10.1146/annurev.ecolsys.110308.120324

[4] Lowe WH, Allendorf FW. What can genetics tell us about population connectivity? Mol Ecol. 2010;19: 3038–3051. 10.1111/j.1365-294X.2010.04688.x 20618697

[5] Taberlet P, Fumagalli L, Wust-Saucy AG, Cosson JF. Comparative phylogeography and postglacial colonization routes in Europe. Mol Ecol. 1998;7: 453–464. 9628000 10.1046/j.1365-294x.1998.00289.x

[6] Hewitt GM. Post-glacial re-colonization of European biota. Biol J Linn Soc Lond. 1999;68: 87–112.

[7] Schmitt T. Molecular biogeography of Europe: Pleistocene cycles and postglacial trends. Front Zool. 2007;4: 11 10.1186/1742-9994-4-11 17439649 PMC1868914

[8] Keyghobadi N. The genetic implications of habitat fragmentation for animals. Can J Zool. 2007;85: 1049–1064.

[9] Emel SL, Storfer A. A decade of amphibian population genetic studies: Synthesis and recommendations. Conserv Genet. 2012;13: 1685–1689. 10.1007/s10592-012-0407-1

[10] Orsini L, Vanoverbeke J, Swillen I, Mergeay J, De Meester L. Drivers of population genetic differentiation in the wild: Isolation by dispersal limitation, isolation by adaptation and isolation by colonization. Mol Ecol. 2013;22: 5983–5999. 10.1111/mec.12561 24128305

[11] Kolbe JJ, Glor RE, Schettino LRG, Lara AC, Larson A, Losos JB. Genetic variation increases during biological invasion by a Cuban lizard. Nature. 2004;431: 177–181. 15356629 10.1038/nature02807

[12] Miller N, Estoup A, Toepfer S, Bourguet D, Lapchin L, Derridj S, et al Multiple transatlantic introductions of the western corn rootworm. Science. 2005;310: 992–992. 16284172 10.1126/science.1115871

[13] Lombaert E, Guillemaud T, Cornuet JM, Malausa T, Facon B, Estoup A. Bridgehead effect in the worldwide invasion of the biocontrol harlequin ladybird. PLoS ONE. 2010;5: e9743 10.1371/journal.pone.0009743 20305822 PMC2840033

[14] Krehenwinkel H, Tautz D. Northern range expansion of European populations of the wasp spider *Argiope bruennichi* is associated with global warming-correlated genetic admixture and population-specific temperature adaptations. Mol Ecol. 2013;22: 2232–2248. 10.1111/mec.12223 23496675

[15] Hedrick PW. Conservation genetics: where are we now? Trends Ecol Evol. 2001;16: 629–636.

[16] Carty J, Ljunggvist C, Prest D, Segura C, Zimmering H. How can molecular genetics help us to prioritize taxa for conservation? J. Conserv Biol. 2009;1: 38–45.

[17] Sarre SD, Georges A. Genetics in conservation and wildlife management: A revolution since Caughley. Wildl. Res. 2009;36: 70–80.

[18] Avise JC. Perspective: conservation genetics enters the genomics era. Conserv Genet. 2010;11: 665–669.

[19] Gompert Z. Population genomics as a new tool for wildlife management. Mol Ecol. 2012;21: 1542–1544. 10.1111/j.1365-294X.2012.05471.x 22443425

[20] Lodge DM. Biological invasions: Lessons for ecology. Trends Ecol Evol. 1993;8: 133–137. 10.1016/0169-5347(93)90025-K 21236129

[21] Hewitt G. The genetic legacy of the Quaternary ice ages. Nature. 2000;405: 907–913. 10.1038/35016000 10879524

[22] Davis MB, Shaw RG. Range shifts and adaptive responses to Quaternary climate change. Science. 2001;292: 673–679. 10.1126/science.292.5517.673 11326089

[23] Chen I-C, Hill JK, Ohlemüller R, Roy DB, Thomas CD. Rapid range shifts of species associated with high levels of climate warming. Science. 2011;333: 1024–1026. 10.1126/science.1206432 21852500

[24] Excoffier L, Foll M, Petit RJ. Genetic consequences of range expansions. Annu Rev Ecol Evol Syst. 2009; 40:481–501. 10.1146/annurev.ecolsys.39.110707.173414

[25] Austerlitz F, Jung-Muller B, Godelle B, Gouyon P-H. Evolution of coalescence times, genetic diversity and structure during colonization. Theor Popul Biol. 1997;51: 148–164. 10.1006/tpbi.1997.1302

[26] Ramakrishnan AP, Musial T, Cruzan MB. Shifting dispersal modes at an expanding species’ range margin. Mol Ecol. 2010;19: 1134–1146. 10.1111/j.1365-294X.2010.04543.x 20456225

[27] Banks SAMC, Ling SD, Johnson CR, Piggott MP, Jane E. Genetic structure of a recent climate change-driven range extension. Mol Ecol. 2010;19: 2011–2024. 10.1111/j.1365-294X.2010.04627.x 20406383

[28] Watts PC, Keat S, Thompson DJ. Patterns of spatial genetic structure and diversity at the onset of a rapid range expansion: colonisation of the UK by the small red-eyed damselfly *Erythromma viridulum* . Biol Invasions. 2010;12: 3887–3903.

[29] Garroway CJ, Bowman J, Holloway GL, Malcolm JR, Wilson PJ. The genetic signature of rapid range expansion by flying squirrels in response to contemporary climate warming. Glob Change Biol. 2011;17: 1760–1769.

[30] Buckley J, Butlin RK, Bridle JR. Evidence for evolutionary change associated with the recent range expansion of the British butterfly, *Aricia agestis*, in response to climate change. Mol Ecol. 2012;21: 267–280. 10.1111/j.1365-294X.2011.05388.x 22118243

[31] Swaegers J, Mergeay J, Therry L, Larmuseau MHD, Bonte D, Stoks R. Rapid range expansion increases genetic differentiation while causing limited reduction in genetic diversity in a damselfly. Heredity. 2013;111:422–429. 10.1038/hdy.2013.64 23820582 PMC3806023

[32] Chapron G, Kaczensky P, Linnell JDC, von Arxx M, Huber D, Andrén H, et al Recovery of large carnivores in Europe’s modern human-dominated landscapes. Science. 2014;346: 1517–1519. 10.1126/science.1257553 25525247

[33] Hagen SB, Kopatz A, Aspi J, Kojola I, Eiken HG. Evidence of rapid change in genetic structure and diversity during range expansion in a recovering large terrestrial carnivore. Proc R Soc B. 2015;282: 20150092 10.1098/rspb.2015.0092 25904665 PMC4424642

[34] Ibrahim KM, Nichols RA, Hewitt GM. Spatial patterns of genetic variation generated by different forms of dispersal during range expansion. Heredity. 1996;77: 282–291.

[35] Kopatz A, Eiken HG, Hagen SB, Ruokonen M, Esparza-Salas R, Schregel J, et al Connectivity and population subdivision at the fringe of a large brown bear (*Ursus arctos*) population in North Western Europe. Conserv Genet. 2012;13: 681–692.

[36] Jansson E, Ruokonen M, Kojola I, Aspi J. Rise and fall of a wolf population: Genetic diversity and structure during recovery, rapid expansion and drastic decline. Mol Ecol. 2012;21: 5178–5193. 10.1111/mec.12010 22978518

[37] Jhala Y, Moehlman PD. *Canis aureus* The IUCN Red List of Threatened Species. Version 2015.1; 2015. Available: www.iucnredlist.org. Accessed 21 June 2015.

[38] Trouwborst A, Krofel M, Linnell JDC. Legal implications of range expansions in a terrestrial carnivore: the case of the golden jackal (*Canis aureus*) in Europe. Biodivers Conserv. 2015;24: 2593–2610.

[39] Flagstad Ø, Walker CW, Vilà C, Sundqvist AK, Fernholm B, Hufthammer AK, et al Two centuries of the Scandinavian wolf population: Patterns of genetic variability and migration during an era of dramatic decline. Mol Ecol. 2003;12: 869–880. 12753208 10.1046/j.1365-294x.2003.01784.x

[40] Lucchini V, Galov A, Randi E. Evidence of genetic distinction and long-term population decline in wolves (*Canis lupus*) in the Italian Apennines. Mol Ecol. 2004;13: 523–536. 14871358 10.1046/j.1365-294x.2004.02077.x

[41] Pilot M, Jędrzejewski W, Branicki W, Sidorovich VE, Jędrzejewska B, Stachura K, et al Ecological factors influence population genetic structure of European grey wolves. Mol Ecol. 2006;15: 4533–4553. 17107481 10.1111/j.1365-294X.2006.03110.x

[42] Hindrikson M, Remm J, Männil P, Ozolins J, Tammeleht E, Saarma U. Spatial genetic analyses reveal cryptic population structure and migration patterns in a continuously harvested grey wolf (*Canis lupus*) population in North-Eastern Europe. PLoS ONE. 2013;8: e75765 10.1371/journal.pone.0075765 24069446 PMC3777892

[43] Hellborg L, Walker CW, Rueness EK, Stacy JE, Kojola I, Valdmann H, et al Differentiation and levels of genetic variation in northern European lynx (*Lynx lynx*) populations revealed by microsatellites and mitochondrial DNA analysis. Conserv Genet. 2002;3: 97–111.

[44] Rueness EK, Jorde PE, Hellborg L, Stenseth NC, Ellegren H, Jakobsen KS. Cryptic population structure in a large, mobile mammalian predator: The Scandinavian lynx. Mol Ecol. 2003;12: 2623–2633. 10.1046/j.1365-294X.2003.01952.x 12969466

[45] Gugolz D, Bernasconi MV, Breitenmoser-Würsten C, Wandeler P. Historical DNA reveals the phylogenetic position of the extinct Alpine lynx. J Zool (Lond). 2008;275: 201–208.

[46] Schmidt K, Kowalczyk R, Ozolins J, Männil P, Fickel J. Genetic structure of the Eurasian lynx population in north-eastern Poland and the Baltic states. Conserv Genet. 2009;10: 497–501. 10.1007/s10592-008-9795-7

[47] Ratkiewicz M, Matosiuk M, Kowalczyk R, Konopiński MK, Okarma H, Ozolins J, et al High levels of population differentiation in Eurasian lynx at the edge of the species’ western range in Europe revealed by mitochondrial DNA analyses. Anim Conserv. 2012;15: 603–612.

[48] Taberlet P, Bouvet J. Mitochondrial DNA polymorphism, phylogeography, and conservation genetics of the brown bear *Ursus arctos* in Europe. Proc Biol Sci. 1994;255: 195–200. 8022838 10.1098/rspb.1994.0028

[49] Murtskhvaladze M, Gavashelishvili A, Tarkhnishvili D. Geographic and genetic boundaries of brown bear (*Ursus arctos*) population in the Caucasus. Mol Ecol. 2010;19: 1828–1841. 10.1111/j.1365-294X.2010.04610.x 20345670

[50] Kocijan I, Galov A, Ćetković H, Kusak J, Gomerčić T, Huber D. Genetic diversity of Dinaric brown bears (*Ursus arctos*) in Croatia with implications for bear conservation in Europe. Mamm Biol. 2011;76: 615–621.

[51] Zachos FE, Cirovic D, Kirschning J, Otto M, Hartl GB, Petersen B, et al Genetic variability, differentiation, and founder effect in golden jackals (*Canis aureus*) from Serbia as revealed by mitochondrial DNA and nuclear microsatellite loci. Biochem Genet. 2009;47: 241–250. 10.1007/s10528-009-9221-y 19169806

[52] Fabbri E, Caniglia R, Galov A, Arbanasić H, Lapini L, Bošković I, et al Genetic structure and expansion of golden jackals (*Canis aureus*) in the north-western distribution range (Croatia and eastern Italian Alps). Conserv Genet. 2014;15: 187–199. 10.1007/s10592-013-0530-7

[53] Andersen LW, Harms V, Caniglia R, Czarnomska SD, Fabbri E, Jędrzejewska B, et al Long-distance dispersal of a wolf, *Canis lupus*, in northwestern Europe. Mamm Res. 2015;60: 163–168. 10.1007/s13364-015-0220-6

[54] Randi E. Genetics and conservation of wolves *Canis lupus* in Europe. Mammal Rev. 2011;41: 99–111. 10.1111/j.1365-2907.2010.00176.x

[55] Kühn W. Die dalmatinishen Schakale. Z Säugetierkd. 1935;10: 144–146.

[56] Demeter A, Spassov N. *Canis aureus* Linnaeus, 1758. Schakal, Goldschakal In: Stubbe M, Krapp F, editors. Handbuch der Säugetiere Europas. Raubsäuger. Teil I, Wiesbaden: AULA-Verlag; 1993 pp. 107–138.

[57] Kryštufek B, Tvrtković N. Variability and identity of the jackals (*Canis aureus*) of Dalmatia. Ann Nat Hist Mus Wien. 1990;91B: 7–25.

[58] Malez V. The zooarcheological data as the base of colonizing the Markova cave on the island of Hvar In: 9th Yugoslavian Speleological Congress. Congress Proceedings; 1984 pp. 617–621. (in Croatian).

[59] Sommer R, Benecke N. Late-Pleistocene and early Holocene history of the canid fauna of Europe (Canidae). Mamm Biol. 2005;70: 227–241.

[60] Tóth T, Krecsák L, Szűcs E, Heltai M, Huszár G. Records of the golden jackal (*Canis aureus* Linnaeus, 1758) in Hungary from 1800th until 2007, based on a literature survey. North West J Zool. 2009;5: 386–405.

[61] Myers N, Mittermeier RA, Mittermeier CG, da Fonseca GAB, Kent J. Biodiversity hotspots for conservation priorities. Nature. 2000;403: 853–858. 10706275 10.1038/35002501

[62] Fredholm M, Wintero AK. Variation of short tandem repeats within and between species belonging to the Canidae family. Mamm Genome. 1995;6: 11–18. 7719020 10.1007/BF00350887

[63] Dolf G, Schläpfer J, Gaillard C, Randi E, Lucchini V, Breitenmoser U, et al Differentiation of the Italian wolf and the domestic dog based on microsatellite analysis. Genet Sel Evol. 2000;32: 533–541. 14736381 10.1186/1297-9686-32-5-533 PMC2706877

[64] Francisco LV, Langston AA, Mellersh CS, Neal CL, Ostrander EA. A class of highly polymorphic tetranucleotide repeats for canine genetic mapping. Mamm Genome. 1996;7: 359–362. 8661717 10.1007/s003359900104

[65] Ostrander EA, Sprague GF, Rine J. Identification and characterization of dinucleotide repeat (CA)n markers for genetic mapping in dog. Genomics. 1993;16: 207–213. 8486359 10.1006/geno.1993.1160

[66] Koskinen MT, Hirvonen H, Landry P-A, Primmer CR. The benefits of increasing the number of microsatellites utilized in genetic population studies: an empirical perspective from analyses of grayling (*Thymallus thymallus*) evolutionary relationships. Hereditas. 2004;141: 61–67. 15383073 10.1111/j.1601-5223.2004.01804.x

[67] Nei M, Roychoudhury AK. Sampling variances of heterozygosity and genetic distance. Genetics. 1974;76: 379–390. 4822472 10.1093/genetics/76.2.379 PMC1213072

[68] Peakall R, Smouse PE. GenAlEx V5: Genetic Analysis in Excel. Population genetic software for teaching and research. 2001; Available: http://www.anu.ed.au/BoZo/GenAlEx/. 10.1093/bioinformatics/bts460 PMC3463245 22820204

[69] Goudet J. FSTAT V2.9.3, a program to estimate and test gene diversities and fixation indices. 2001; Available: http://www.unil.ch/izea/softwares/fstat.htlm.

[70] Raymond M, Rousset F. GENEPOP (version 1.2): population genetics software for exact tests and ecumenicism. J Hered. 1995;86: 248–249.

[71] Rousset F. Genepop’007: a complete reimplementation of the Genepop software for Windows and Linux. Mol Ecol Resour. 2008;8: 103–106. 10.1111/j.1471-8286.2007.01931.x 21585727

[72] Petit RJ, El Mousadik A, Pons O. Identifying populations for conservation on the basis of genetic markers. Conserv Biol. 1998;12: 844–855.

[73] Weir BS, Cockerham CC. Estimating F-statistics for the analysis of population structure. Evolution. 1984;38: 1358–1370. 28563791 10.1111/j.1558-5646.1984.tb05657.x

[74] Pritchard JK, Stephens M, Donnelly P. Inference of population structure using multilocus genotype data. Genetics. 2000;155: 945–959. 10835412 10.1093/genetics/155.2.945 PMC1461096

[75] Evanno G, Regnaut S, Goudet J. Detecting the number of clusters of individuals using the software STRUCTURE: a simulation study. Mol Ecol. 2005;14: 2611–2620. 15969739 10.1111/j.1365-294X.2005.02553.x

[76] Earl DA, vonHoldt BM. STRUCTURE HARVESTER: a website and program for visualizing STRUCTURE output and implementing the Evanno method. Conserv Genet Resour. 2012;4: 359–361.

[77] Jakobsson M, Rosenberg NA. CLUMPP: a cluster matching and permutation program for dealing with label switching and multimodality in analysis of population structure. Bioinformatics. 2007;23: 1801–1806. 17485429 10.1093/bioinformatics/btm233

[78] Rosenberg NA. DISTRUCT, a program for the graphical display of population structure, Mol Ecol Notes. 2004;4: 137–138.

[79] Corander J, Marttinen P. Bayesian identification of admixture events using multi-locus molecular markers. Mol Ecol. 2006;15: 2833–2843. 16911204 10.1111/j.1365-294X.2006.02994.x

[80] Corander J, Marttinen P, Sirén J, Tang J. Enhanced Bayesian modelling In BAPS software for learning genetic structures of populations. BMC Bioinformatics. 2008;9: 539 10.1186/1471-2105-9-539 19087322 PMC2629778

[81] Corander J, Sirén J, Arjas E. Bayesian spatial modelling of genetic population structure. Comput Stat. 2008;23: 111–129.

[82] Patterson N, Price AL, Reich D. Population structure and eigenanalysis. PLoS Genet. 2006;2: e190 17194218 10.1371/journal.pgen.0020190 PMC1713260

[83] McVean G. A genealogical interpretation of principal components analysis. PLoS Genet. 2009;5: e1000686 10.1371/journal.pgen.1000686 19834557 PMC2757795

[84] François O, Durand E. Spatially explicit Bayesian clustering models in population genetics. Mol Ecol Resour. 2010;10: 773–784. 10.1111/j.1755-0998.2010.02868.x 21565089

[85] Jombart T. ADEGENET: a R package for the multivariate analysis of genetic markers. Bioinformatics. 2008;24: 1403–1405. 10.1093/bioinformatics/btn129 18397895

[86] Hall TA. BioEdit: a user-friendly biological sequence alignment editor and analysis program for Windows 95/98/NT. Nucleic Acids Symp Ser. 1999;41: 95–98.

[87] Librado P, Rozas J. DNAsp v5: A software for comprehensive analysis of DNA polymorphism data. Bioinformatics. 2009;25: 1451–1452. 10.1093/bioinformatics/btp187 19346325

[88] Excoffier L, Lischer HEL. Arlequin suite ver 3.5: a new series of programs to perform population genetics analyses under Linux and Windows. Mol Ecol Res. 2010;10: 564–567. 10.1111/j.1755-0998.2010.02847.x 21565059

[89] Hudson RR, Boos DD, Kaplan NL. A statistical test for detecting geographic subdivision. Mol Biol Evol. 1992;9: 138–151. 1552836 10.1093/oxfordjournals.molbev.a040703

[90] Bandelt H-J, Forster P, Röhl A. Median-joining networks for inferring intraspecific phylogenies. Mol Biol Evol. 1999;16: 37–48. 10331250 10.1093/oxfordjournals.molbev.a026036

[91] Rueness EK, Asmyhr MG, Sillero-Zubiri C, Macdonald DW, Bekele A, Atickem A, et al The cryptic African wolf: *Canis aureus lupaster* is not a golden jackal and is not endemic to Egypt. PLoS ONE. 2011;6: e16385 10.1371/journal.pone.0016385 21298107 PMC3027653

[92] Randi E, Lucchini V, Christensen MF, Mucci N, Funk SM, Dolf G, et al Mitochondrial DNA variability in Italian and east European wolves: Detecting the consequences of small population size and hybridization. Conserv Biol. 2000;14: 464–473.

[93] Kowalczyk R, Kołodziej-Sobocińska M, Ruczyńska I, Wójcik JM. Range expansion of the golden jackal (*Canis aureus*) into Poland: first records. Mamm Res. 2015;60: 411–414.

[94] Pilot M, Dąbrowski MJ, Hayrapetyan V, Yavruyan EG, Kopaliani N, Tsingarska E, et al Genetic variability of the grey wolf *Canis lupus* in the Caucasus in comparison with Europe and the Middle East: Distinct or intermediary population? PLoS ONE. 2014;9: e93828 10.1371/journal.pone.0093828 24714198 PMC3979716

[95] Aggarwal RK, Kivisild T, Ramadevi J, Singh L. Mitochondrial DNA coding region sequences support the phylogenetic distinction of two Indian wolf species. J Zool Syst Evol Res. 2007;45: 163–172.

[96] Frankham R. Do island populations have less genetic variation than mainland populations? Heredity. 1997;78: 311–327. 9119706 10.1038/hdy.1997.46

[97] Mason RAB, Browning TL, Eldridge MDB. Reduced MHC class II diversity in island compared to mainland populations of the black-footed rock-wallaby (*Petrogale lateralis lateralis*). Conserv Genet. 2011;12: 91–103.

[98] Wang S, Zhu W, Gao X, Li X, Yan S, Liu X, et al Population size and time since island isolation determine genetic diversity loss in insular frog populations. Mol Ecol. 2014;23: 637–648. 10.1111/mec.12634 24351057

[99] Aspi J, Roininen E, Kiiskilä J, Ruokonen M, Kojola I, Bljudnik L, et al Genetic structure of the northwestern Russian wolf populations and gene flow between Russia and Finland. Conserv Genet. 2009;10: 815–826.

[100] Baltrūnaitė L, Balčiauskas L, Åkesson M. The genetic structure of the Lithuanian wolf population. Cent Eur J Biol. 2013;8: 440–447.

[101] Wandeler P, Funk SM, Largiadèr CR, Gloor S, Breitenmoser U. The city-fox phenomenon: Genetic consequences of a recent colonization of urban habitat. Mol Ecol. 2003;12: 647–656. 12675821 10.1046/j.1365-294x.2003.01768.x

[102] Galov A, Sindičić M, Andreanszky T, Čurković S, Dežđek D, Slavica A, et al High genetic diversity and low population structure in red foxes (*Vulpes vulpes*) from Croatia. Mamm Biol. 2014;79: 77–80.

[103] Cohen TM, King R, Dolev A, Boldo A, Lichter-Peled A, Kahila Bar-Gal G. Genetic characterization of populations of the golden jackal and the red fox in Israel. Conserv Genet. 2013;14: 55–63.

[104] Koepfli K-P, Pollinger J, Godinho R, Robinson J, Lea A, Hendricks S, et al Genome-wide evidence reveals that African and Eurasian golden jackals are distinct species. Curr Biol. 2015;25: 2158–2165. 10.1016/j.cub.2015.06.060 26234211

[105] Arnold J, Humer A, Heltai M, Murariu D, Spassov N, Hackländer K. Current status and distribution of golden jackals *Canis aureus* in Europe. Mamm Rev. 2012;42: 1–11.

[106] Edwards CJ, Soulsbury CD, Statham MJ, Ho SYW, Wall D, Dolf G, et al Temporal genetic variation of the red fox, *Vulpes vulpes*, across western Europe and the British Isles. Quat Sci Rev. 2012;57: 95–104. 24068852 10.1016/j.quascirev.2012.10.010 PMC3778924

[107] Djan M, Maletić V, Trbojević I, Popović D, Veličković N, Burazerović J, et al Genetic diversity and structuring of the grey wolf population from the Central Balkans based on mitochondrial DNA variation. Mamm Biol. 2014;79: 277–282. 10.1016/j.mambio.2014.03.001

[108] Fabbri E, Caniglia R, Kusak J, Galov A, Gomerčić T, Arbanasić H, et al Genetic structure of expanding wolf (*Canis lupus*) populations in Italy and Croatia, and the early steps of the recolonization of the Eastern Alps. Mamm Biol. 2014;79: 138–148. 10.1016/j.mambio.2013.10.002

[109] Pilot M, Jędrzejewski W, Sidorovich VE, Meier-Augenstein W, Hoelzel AR. Dietary differentiation and the evolution of population genetic structure in a highly mobile carnivore. PLoS ONE. 2012;7: e39341 10.1371/journal.pone.0039341 22768075 PMC3387138

[110] Jędrzejewski W, Niedziałkowska M, Hayward MW, Goszczyński J, Jędrzejewska B, Borowik T, et al Prey choice and diet of wolves related to ungulate communities and wolf subpopulations in Poland. J Mammal. 2012;93: 1480–1492.

[111] Vuletić-Vukasović V. Čagalj na Korčuli. Dubrovnik: Štamparija Degiulli i dr; 1908.

[112] Giannatos G, Marinos Y, Maragou P, Katsadorakis G. The status of the golden jackal (*Canis aureus* L.) in Greece. Belg J Zool. 2005;135: 145–149.

[113] Banea O. Jackals in West Estonia. 2013 Mar 1. In: GOJAGE blog [Internet] [about 18 screens]. 2013; Available: http://goldenjackalaround.blogspot.com/2013/03/golden-jackal-survey-in-w-estonia.html. Accessed 13 January 2015.

[114] Toom M. Šaakali (*Canis aureus* L.) areaali laienemine Euroopas viimastel aastakümnetel [Jackal range expansion in Europe]. BA Thesis, Estonian University of Life Sciences, Tartu. 2014. (in Estonian with English abstract).

[115] Levickaitė R. Lietuvoje sumedžiotas pirmasis šakalas—didžiulės bėdos pranašas. 2015; Available: http://grynas.delfi.lt/gamta/lietuvoje-sumedziotas-pirmasis-sakalas-didziules-bedos-pranasas.d?id=67703144. Accessed 12 May 2015 (in Lithuanian).

[116] Roženko N, Volokh A. The golden jackal (*Canis aureus* L, 1758) as a new species in the fauna of Ukraine. Beitr Jagd-Wildforsch. 2010;35: 237–246.

[117] STV. Žitel’ iz derevni Tomašovka Brestskoj oblasti podstrelil šakala. Telekanal STV [Internet]. Belarus. 2012; Available: http://www.ctv.by. Accessed 7 March 2015. (in Russian).

[118] Thibault I, Bernatchez L, Dodson JJ. The contribution of newly established populations to the dynamics of range expansion in a one-dimensional fluvial-estuarine system: rainbow trout (*Oncorhynchus mykiss*) in Eastern Quebec. Divers Distrib. 2009;15: 1060–1072.

[119] Šálek M, Červinka J, Banea CO, Krofel M, Ćirović D, Selanec I, et al Population densities and habitat use of the golden jackal (*Canis aureus*) in farmlands across the Balkan Peninsula. Eur J Wildl Res. 2014;60:193–200.

[120] Kareiva P. Population dynamics in spatially complex environments: theory and data. Philos Trans R Soc Lond B Biol Sci. 1990;330: 175–190.

[121] Mehora M. Šakali v Sloveniji. Lovec. 1953;36: 470.

[122] Brelih S. Šakali (*Canis aureus* L.) na ozemlju Slovenije. Biol Vest. 1955; 4: 56–58.

[123] Kryštufek B, Tvrtković N. Range expansion by Dalmatian jackal population in the 20th-century (*Canis aureus* Linnaeus, 1758). Folia Zool. 1990;39: 291–296.

[124] Möckel R. Ein Goldschakal (*Canis aureus*) in Südbrandenburg: Erstnachweis für Deutschland. Säugetierkd Informationen. 2000;4: 477–481.

[125] Weingarth K, Gahbauer M, Heurich M, Müller J, Leibl F. Second record of a golden jackal (*Canis aureus*) in Germany. Säugetierkd Informationen. 2012;8: 443–446.

[126] KORA Erster Goldschakal-Nachweis in der Schweiz. KORA News. 8 Sep 2012. 2012; Available: http://www.kora.ch/index.php?id=214andL=1andtx_ttnews%5Btt_news%5D=408andcHash=be9f32a37b98a1803257ca8cba134657. Accessed 13 January 2015.

[127] Žila S. Šakal v Ukraїnі: stratiti či pomiluvati? 2014; Available: http://www.hunt-fish.com.ua/article.htm?ident=c3f4fe7219d91b0. Accessed 20 January 2015. (in Ukrainian).

[128] Stratford J. Golden jackal in Lithuania, a consideration of its arrival, impact and status. Zool Ecol. 2015; 1 9 2015. 10.1080/21658005.2015.1073894

[129] Convention on Biological Diversity. What are Invasive Alien Species? 2015. Available: https://www.cbd.int/invasive/WhatareIAS.shtml. Accessed 8 September 2015.

[130] Szabó L, Heltai M, Szűcs E, Lanszki J, Lehoczki R. Expansion range of the golden jackal in Hungary between 1997 and 2006. Mammalia. 2009;73: 307–311.

[131] Heltai M, Ćirović D, Szabó L, Penezić A, Nagyapáti N, Kurys A, et al. Golden jackal: opinions versus facts—experiences from Serbia and Hungary. In: 2nd International Symposium on Hunting “Мodern aspects of sustainable management of game population”, Zemun-Belgrade, Serbia, 22–24. June, 2012; 2012. pp. 13–20.

[132] Stoyanov S. Golden jackal (*Canis aureus*) in Bulgaria: current status, distribution, demography and diet. In: 2nd International Symposium on Hunting “Мodern aspects of sustainable management of game population”, Zemun-Belgrade, Serbia, 22–24. June, 2012; 2012. pp. 48–56.

[133] Bošković I, Šperanda M, Florijančić T, Šprem N, Ozimec S, Degmečić D, et al Dietary habits of the golden jackal (*Canis aureus* L.) in the Eastern Croatia. Agric Conspec Sci. 2013;78: 245–248.

[134] Lanszki J, Kurys A, Heltai M, Csányi S, Ács K. Diet composition of the golden jackal in an area of intensive big game management. Ann Zool Fenn. 2015; 52: 243–255.

[135] Giannatos G. Population status and Conservation Action Plan for the golden jackal (*Canis aureus*) in Greece. Athens: World Wildlife Fund; 2004.

[136] Ćirović D, Penezić A, Milenković M, Paunović M. Winter diet composition of the golden jackal (*Canis aureus* L., 1758) in Serbia. Mamm Biol. 2014;79: 132–137.

[137] Spassov N. The position of jackals in the *Canis* genus and life-history of the golden jackal (*Canis aureus* L.) in Bulgaria and on the Balkans. Hist Nat Bulg. 1989:1; 44–56.

[138] Mihelič M, Krofel M. New records of the golden jackal (*Canis aureus* L.) in the upper Soča valley, Slovenia. Nat Slov. 2012;14: 51–63.

[139] Yom-Tov Y, Ashkenazi S, Viner O. Cattle predation by the golden jackal *Canis aureus* in the Golan Heights, Israel. Biol Conserv. 1995;73: 19–22.

[140] Ćirović D, Penezić A, Pavlović I, Kulišić Z, Ćosić N, Burazerović J, Maletić V. First records of *Dirofilaria repens* in wild canids from the region of Central Balkan. Acta Vet Hung. 2014;62: 481–488. 10.1556/AVet.2014.021 25410390

[141] Penezić A, Selaković S, Pavlović I, Ćirović D. First findings and prevalence of adult heartworms (*Dirofilaria immitis*) in wild carnivores from Serbia. Parasitol Res. 2014;113: 3281–3285. 10.1007/s00436-014-3991-9 24951168

[142] Ćirović D, Pavlović I, Penezić A. Intestinal helminth parasites of the grey wolf (*Canis lupus* L.) in Serbia. Acta Vet Hung. 2015;63: 189–198. 10.1556/AVet.2015.016 26051257

[143] Ćirović D, Pavlović I, Penezić A, Kulisić Z, Selaković S. Levels of infection of intestinal helminth species in the golden jackal *Canis aureus* from Serbia. J Helminthol. 2015;89: 28–33. 10.1017/S0022149X13000552 23941681

[144] Linnell JDC, Salvatori V, Boitani L. Guidelines for population level management plans for large carnivores in Europe. A Large Carnivore Initiative for Europe report prepared for the European Commission; 2008 (contract 070501/2005/424162/MAR/B2).

