# Supplementary material for: A European Concern? Genetic Structure and Expansion of Golden Jackals (Canis aureus) in Europe and the Caucasus
Source: PLoS One. 2015 Nov 5;10(11):e0141236. doi: 10.1371/journal.pone.0141236 (PMC4634961; doi:10.1371/journal.pone.0141236)
Supplement: S1 File — Table A, List of examined specimens including specimen number, sex, locality information, date, geographical coordinates, and mtDNA haplotype designation. Table B, Microsatellite genotypes. Table C, Per locus genetic diversity in 97 samples of golden jackal. Table D, Per region genetic diversity estimated based on polymorphisms in 15 microsatellite loci. (DOC) [file pone.0141236.s001.doc]

**Supplementary Information (S1)**

**Table A. List of examined specimens including specimen number, sex, locality information, date, geographical coordinates and mtDNA haplotype designation.**

| Spec. Id. | Sex | Country/  Region | Place | Date | Geographical coordinates | | Haplotype |
| --- | --- | --- | --- | --- | --- | --- | --- |
|  |  |  |  |  | Longitude (ºE) | Latitude (ºN) |  |
| 5814 | ? | Armenia | Vardenis, Gegarkunik province | 27.07.2011 | 45.720 | 40.180 | H3 |
| 6355 | ♂ | Armenia | Martuninskij region, Gegarkunik province | 16.10.2011 | 45.300 | 40.140 | - |
| 7021 | ♀ | Armenia | Gegarkunik region, Dilijan, Shorja | 16.01.2012 | 45.270 | 40.500 | H3 |
| 8207 | ? | Croatia | Pelješac Peninsula | 8−9.11.2012 | 17.500 | 42.888 | H1 |
| 8208 | ? | Croatia | Pelješac Peninsula | 8−9.11.2012 | 17.362 | 42.943 | H1 |
| 7869 | ♀ | Estonia | Lääne country, Lihula municipality | 28.02.2013 | 26.140 | 59.220 | H1 |
| 8267 | ♂ | Estonia | Ida-Viru county, Lüganuse municipality | 06.08.2013 | 24.488 | 58.101 | H1 |
| 8887 | ♀ | Estonia | Pärnu county, Häädemeeste municipality | 09.12.2013 | 23.535 | 58.697 | - |
| 8888 | ♂ | Estonia | Lääne county, Hanila municipality | 29.01.2014 | 23.579 | 58.702 | H1 |
| 8919 | ? | Georgia | Great Caucasus | Winter 2007 | 43.331 | 42.444 | H1 |
| 8920 | ? | Georgia | Lesser Caucasus | Summer 2009 | 42.420 | 42.150 | H1 |
| 8921 | ? | Georgia | Colchis lowland | Spring 2013 | 41.523 | 42.125 | H1 |
| 8922 | ? | Georgia | Colchis lowland | Winter 2014 | 41.441 | 42.221 | H1 |
| 8923 | ? | Georgia | Colchis lowland | Spring 2014 | 41.482 | 42.238 | H1 |
| 8960 | ? | Greece | Peloponnese, road from Astros to Leonidio, ca. 1.5 km SE from Astros | 25.11.2012 | 22.736 | 37.394 | H4 |
| 8961 | ? | Greece | Peloponnese, ca. 1.5 km SE from Astros | 25.11.2012 | 22.737 | 37.394 | H1 |
| 8962 | ? | Greece | Peloponnese, road from Astros to Tripolis, ca. 16 km W-NW from Astros | 19.02.2013 | 22.547 | 37.435 | H1 |
| 8963 | ? | Greece | Peloponnese, road from Astros to Leonidio, ca. 2 km SE from Astros | 01.06.2013 | 22.741 | 37.387 | H4 |
| 8964 | ? | Greece | Peloponnese, road from Astros to Agios Petros, 3.3 SW km from Astros | 02.06.2013 | 22.695 | 37.382 | H4 |
| 8965 | ? | Greece | Peloponnese, road from Astros to Tripolis, ca. 5.6 km NW from Astros | 26.08.2014 | 22.672 | 37.431 | H1 |
| 8967 | ? | Greece | Peloponnese, road from Agios Andreas to Kastanitsa, ca. 7.6 km S from Astros | 01.10.2014 | 22.736 | 37.335 | H4 |
| 8968 | ? | Greece | Peloponnese, road from Astros to Tripolis, ca. 6.3 km NW from Astros | 15.10.2014 | 22.664 | 37.432 | H1 |
| 8971 | ? | Greece | Peloponnese, road from Astros to Tripolis, ca. 8 km NW from Astros | 05.11.2014 | 22.646 | 37.438 | H1 |
| 8972 | ? | Greece | Samos Island | 01.01.2012 | 26.815 | 37.732 | H3 |
| 8973 | ♀ | Greece | Samos Island | 06.11.2014 | 26.770 | 37.755 | H3 |
| 8976 | ? | Greece | Samos Island | 14.12.2010 | 26.754 | 37.687 | H2 |
| 8977 | ♀ | Greece | Samos Island | 14.03.2014 | 26.815 | 37.732 | H2 |
| 8978 | ♂ | Greece | Samos Island | 17.12.2013 | 26.806 | 37.696 | H3 |
| 8979 | ♀ | Greece | Samos Island | 14.04.2013 | 26.815 | 37.732 | H2 |
| 8980 | ♀ | Greece | Samos Island | 18.09.2012 | 26.815 | 37.732 | H2 |
| 8981 | ♀ | Greece | Samos Island | 11.02.2013 | 26.750 | 37.722 | H2 |
| 8982 | ? | Greece | Samos Island | 16.07.2013 | 26.815 | 37.732 | H2 |
| 8985 | ♀ | Greece | Samos Island | 23.12.2013 | 26.815 | 37.732 | H2 |
| 8986 | ? | Greece | Chalkidiki Penninsula, near Stratoniki | 22.10.2014 | 23.062 | 40.554 | H1 |
| 9064 | ? | Greece | N. Peloponnesus |  | 22.254 | 38.037 | H1 |
| 9065 | ? | Greece | N. Peloponnesus |  | 22.153 | 38.011 | H1 |
| 8852 | ♀ | Hungary | Lábod | 27.09.2006 | 17.470 | 46.204 | H1 |
| 8853 | ♀ | Hungary | Velencei tó | 19.08.2008 | 18.582 | 47.219 | H1 |
| 8854 | ♀ | Hungary | Kétújfalu | 04.03.2012 | 17.714 | 45.966 | H1 |
| 8855 | ♀ | Hungary | kiskunhalas | 10.01.2012 | 19.488 | 46.432 | H1 |
| 8856 | ♀ | Hungary | Ócsa | 28.07.2011 | 19.226 | 47.294 | H1 |
| 8857 | ♀ | Hungary | Paks | - | 18.862 | 46.625 | H1 |
| 8858 | ♂ | Hungary | Sukoró | 07.08.2013 | 18.601 | 47.240 | H1 |
| 8859 | ♂ | Hungary | *Unknown* (centered on Hungary) | 15.07.2013 | 17.400 | 46.900 | H1 |
| 8860 | ♀ | Hungary | Hajós | 22.08.2013 | 19.134 | 46.320 | H1 |
| 8861 | ♂ | Hungary | Lábod | - | 17.470 | 46.204 | H1 |
| 9225 | ? | Lithuania | Šakiai district, Lekėčiai | Feb. 2015 | 23.579 | 58.702 | H1 |
| 5732 | ♀ | Mountainous Karabakh | Qelbajar | 09.07.2011 | 46.050 | 40.010 | H1 |
| 5733 | ♂ | Mountainous Karabakh | Qelbajar | 09.07.2011 | 46.050 | 40.010 | H1 |
| 5737 | ♀ | Mountainous Karabakh | Agdam | 04.06.2011 | 46.946 | 40.004 | H3 |
| 5787 | ? | Mountainous Karabakh | S.N. Oratag | 11.05.2011 | 46.540 | 40.130 | H1 |
| 8322 | ♀ | Mountainous Karabakh | Carcar region | 02.10.2012 | 47.027 | 39.746 | H1 |
| 8457 | ♂ | Mountainous Karabakh | Hadrut | 03.10.2013 | 47.029 | 39.515 | H1 |
| 7870 | ? | Romania | Cheveresu Mare | Feb. 2011 | 21.499 | 45.710 | H1 |
| 7871 | ? | Romania | Cheveresu Mare | Feb. 2011 | 21.499 | 45.710 |  |
| 7872 | ? | Romania | Cheveresu Mare | Feb. 2011 | 21.499 | 45.710 | H1 |
| 7873 | ? | Romania | Petru Rares | Feb. 2011 | 25.818 | 44.118 | H1 |
| 7874 | ? | Romania | Varasti | Feb. 2011 | 26.985 | 44.206 | H1 |
| 8206 | ? | Serbia | Vranje | 01.05.2011 | 21.934 | 42.547 |  |
| 8609 | ♂ | Serbia | Vojvodina, Pećinci | 5.10.2013 | 19.961 | 44.898 | H1 |
| 8610 | ♂ | Serbia | Vojvodina, Pećinci | 17.07.2011 | 19.961 | 44.898 | H1 |
| 8611 | ♂ | Serbia | Vojvodina, Pećinci | 6.01.2013 | 19.961 | 44.898 | H1 |
| 8612 | ♂ | Serbia | Vojvodina, Pećinci | 25.07.2013 | 19.961 | 44.898 | H1 |
| 8613 | ♂ | Serbia | Vojvodina, Pećinci | 6.01.2013 | 19.961 | 44.898 | H1 |
| 8614 | ♂ | Serbia | Vojvodina, Pećinci | 24.02.2013 | 19.961 | 44.898 | H1 |
| 8615 | ♀ | Serbia | Vojvodina, Pećinci | 1.11.2013 | 19.961 | 44.898 | H1 |
| 8616 | ♀ | Serbia | Vojvodina, Pećinci | 18.11.2013 | 19.961 | 44.898 | H1 |
| 8617 | ♂ | Serbia | Vojvodina, Novi Kneževac | 5.11.2009 | 20.109 | 46.048 | H1 |
| 8618 | ♂ | Serbia | Vojvodina, Novi Kneževac | 10.01.2009 | 20.109 | 46.048 | H1 |
| 8619 | ♀ | Serbia | Vojvodina, Novi Kneževac | 5.11.2009 | 20.109 | 46.048 | H1 |
| 8620 | ♀ | Serbia | Vojvodina, Titel | 10.12.2012 | 20.300 | 45.200 | H1 |
| 8621 | ♀ | Serbia | Vojvodina, Titel | 4.01.2013 | 20.300 | 45.200 | H1 |
| 8622 | ♀ | Serbia | Vojvodina, Titel | 16.02.2013 | 20.300 | 45.200 | H1 |
| 8623 | ♀ | Serbia | Vojvodina, Borča | 31.08.2013 | 20.467 | 44.867 | H1 |
| 8624 | ♂ | Serbia | Vojvodina, Borča | 30.05.2013 | 20.467 | 44.867 | H1 |
| 8625 | ♀ | Serbia | Vojvodina, Borča | 31.08.2013 | 20.467 | 44.867 | H1 |
| 8626 | ♂ | Serbia | Vojvodina, Borča | 2.01.2010 | 20.467 | 44.867 | H1 |
| 8627 | ♀ | Serbia | Vojvodina, Borča | 2.10.2010 | 20.467 | 44.867 | H1 |
| 8628 | ♂ | Serbia | Vojvodina, Dobanovci | 27.11.2011 | 20.230 | 44.830 | H1 |
| 8629 | ♀ | Serbia | Vojvodina, Dobanovci | 9.09.2013 | 20.230 | 44.830 | H1 |
| 8630 | ♂ | Serbia | Vojvodina, Dobanovci | 20.06.2013 | 20.230 | 44.830 | H1 |
| 8631 | ♀ | Serbia | Vojvodina, Dobanovci | 3.11.2013 | 20.230 | 44.830 | H1 |
| 8632 | ♀ | Serbia | Vojvodina, Dobanovci | 9.09.2013 | 20.230 | 44.830 | H1 |
| 8209 | ? | Slovenia | Ljubljansko barje | 16.08.2010 | 14.415 | 45.977 | H1 |
| 8933 | ♂ | Slovenia | Ljubljansko barje | 10.09.2014 | 14.476 | 46.025 | H1 |
| 8924 | ♂ | Ukraina | Kherson district, Genichesky region, Biruchiy Peninsula | 06.12. 2012 | 35.042 | 46.721 | H1 |
| 8925 | ♀ | Ukraina | Kherson district, Genichesky region, Biruchiy Peninsula | 06.12. 2012 | 35.042 | 46.721 | H1 |
| 8927 | ♂ | Ukraina | Kherson district, Genichesky region, Biruchiy Peninsula | 26.12. 2012 | 35.042 | 46.721 | H4 |
| 8597 | ♂ | Ukraine | Odeski district, Kilysky region, Kislica village | 26.12.2013 | 29.073 | 45.389 | H1 |
| 8598 | ♂ | Ukraine | Odeski district, Kilysky region, Kislica village | 28.12.2013 | 29.073 | 45.389 | H1 |
| 8599 | ♀ | Ukraine | Odeski district, Kilysky region, Vasilivka village | 22.03.2009 | 29.179 | 45.493 | H1 |
| 8600 | ♂ | Ukraine | Odeski district, Bilaivsky region, Majki village | Dec. 2010 | 30.134 | 46.412 | H1 |
| 8601 | ♂ | Ukraine | Odeski district, Ivanivsky region, Bilka village | Feb. 2011 | 30.422 | 46.904 | H1 |
| 8602 | ♂ | Ukraine | Kherson district, Curupinsky region, Kozachi Lageri village | 22.08.2011 | 32.975 | 46.718 | H1 |
| 8605 | ♀ | Ukraine | Zaporizhzha district, Zaporizhzha region, Krutoyarivske forest region | 03.12.2008 | 35.067 | 47.727 | H1 |
| 8607 | ♀ | Ukraine | Kherson district, Genichesky region, Biruchiy Peninsula | 19.03.2011 | 35.071 | 46.123 | H1 |
| 8608 | ♂ | Ukraine | Poltavska district, Каrlivsky region,  Bilukhivka village | 22.12. 2012 | 35.294 | 49.588 | H1 |

**Table B. Microsatellite genotypes.** The ‘ID’ column is an individual identifier. Subsequent columns provide genotypes for the 15 autosomal microsatellite loci as nominal sizes in base pairs. For other details see S1 Table.

| **ID** | **CPH4** | | **CPH5** | | **CPH8** | | **CPH12** | | **CPH6** | | **CPH9** | | **CPH22** | | **FH2004** | | **FH2088** | | **FH2096** | | **FH2137** | | **FH2140** | | **CXX.213** | | **C09.250** | | **C20.253** | |
| --- | --- | --- | --- | --- | --- | --- | --- | --- | --- | --- | --- | --- | --- | --- | --- | --- | --- | --- | --- | --- | --- | --- | --- | --- | --- | --- | --- | --- | --- | --- |
| 7870 | 140 | 142 | 120 | 120 | 200 | 202 | 200 | 200 | 127 | 127 | 145 | 151 | 121 | 121 | 310 | 318 | 96 | 96 | 102 | 102 | 154 | 182 | 111 | 111 | 160 | 164 | 117 | 129 | 99 | 101 |
| 7871 | 140 | 152 | 120 | 120 | 198 | 202 | 200 | 200 | 127 | 127 | 145 | 151 | 121 | 121 | 302 | 318 | 92 | 96 | 94 | 102 | 154 | 154 | 123 | 123 | 160 | 164 | 129 | 129 | 93 | 95 |
| 7872 | 142 | 152 | 120 | 120 | 198 | 198 | 202 | 202 | 125 | 127 | 151 | 151 | 121 | 121 | 310 | 318 | 92 | 92 | 82 | 102 | 154 | 154 | 123 | 123 | 160 | 160 | 129 | 129 | 93 | 101 |
| 7873 | 142 | 152 | 120 | 120 | 198 | 202 | 200 | 200 | 125 | 127 | 151 | 151 | 121 | 121 | 310 | 318 | 92 | 92 | 82 | 102 | 178 | 202 | 123 | 123 | 160 | 164 | 129 | 131 | 101 | 101 |
| 7874 | 142 | 152 | 120 | 122 | 202 | 202 | 200 | 200 | 127 | 127 | 151 | 151 | 119 | 119 | 310 | 318 | 92 | 96 | 102 | 102 | 154 | 154 | 111 | 123 | 160 | 160 | 117 | 131 | 95 | 99 |
| 8207 | 140 | 142 | 120 | 120 | 200 | 202 | 200 | 200 | 123 | 127 | 151 | 151 | 121 | 121 | 306 | 310 | 92 | 96 | 82 | 94 | 150 | 154 | 123 | 131 | 162 | 164 | 117 | 117 | 95 | 101 |
| 8208 | 142 | 152 | 120 | 120 | 202 | 202 | 200 | 200 | 127 | 127 | 141 | 151 | 119 | 121 | 310 | 318 | 96 | 96 | 94 | 102 | 150 | 150 | 111 | 131 | 160 | 162 | 117 | 117 | 101 | 101 |
| 8209 | 152 | 152 | 120 | 122 | 202 | 202 | 200 | 202 | 123 | 127 | 151 | 157 | 119 | 121 | 310 | 318 | 96 | 96 | 94 | 102 | 154 | 182 | 111 | 123 | 160 | 160 | 117 | 131 | 95 | 99 |
| 8933 | 142 | 152 | 120 | 122 | 200 | 202 | 200 | 200 | 123 | 123 | 145 | 151 | 121 | 121 | 318 | 318 | 90 | 94 | 94 | 102 | 154 | 182 | 111 | 111 | 158 | 160 | 129 | 129 | 99 | 99 |
| 8597 | 152 | 152 | 120 | 120 | 202 | 202 | 200 | 200 | 123 | 127 | 151 | 151 | 121 | 121 | 290 | 318 | 92 | 96 | 94 | 102 | 178 | 202 | 123 | 131 | 160 | 160 | 117 | 117 | 99 | 107 |
| 8598 | 140 | 142 | 114 | 120 | 202 | 202 | 200 | 202 | 123 | 129 | 145 | 151 | 119 | 121 | 298 | 318 | 96 | 96 | 94 | 102 | 182 | 182 | 123 | 123 | 160 | 162 | 129 | 131 | 95 | 99 |
| 8599 | 140 | 142 | 120 | 122 | 202 | 204 | 200 | 200 | 123 | 125 | 145 | 157 | 121 | 121 | 290 | 310 | 92 | 92 | 94 | 102 | 154 | 154 | 123 | 131 | 160 | 160 | 133 | 133 | 101 | 101 |
| 8600 | 142 | 142 | 120 | 122 | 202 | 202 | 200 | 200 | 123 | 123 | 151 | 157 | 121 | 121 | 306 | 318 | 92 | 96 | 94 | 102 | 178 | 202 | 111 | 131 | 134 | 160 | 129 | 129 | 95 | 109 |
| 8601 | 140 | 152 | 120 | 122 | 202 | 202 | 200 | 200 | 123 | 129 | 151 | 151 | 121 | 121 | 318 | 318 | 92 | 96 | 94 | 102 | 154 | 202 | 131 | 131 | 150 | 160 | 117 | 117 | 93 | 93 |
| 8602 | 140 | 142 | 120 | 122 | 202 | 202 | 200 | 200 | 123 | 127 | 151 | 157 | 119 | 121 | 290 | 310 | 96 | 96 | 102 | 102 | 154 | 182 | 123 | 131 | 160 | 162 | 117 | 129 | 93 | 99 |
| 8605 | 152 | 152 | 120 | 122 | 202 | 202 | 202 | 202 | 127 | 127 | 151 | 151 | 121 | 121 | 318 | 318 | 96 | 96 | 94 | 102 | 178 | 182 | 123 | 123 | 160 | 160 | 129 | 129 | 101 | 101 |
| 8607 | 140 | 140 | 114 | 122 | 202 | 202 | 200 | 200 | 123 | 123 | 145 | 151 | 119 | 121 | 290 | 302 | 92 | 92 | 94 | 102 | 154 | 162 | 119 | 123 | 160 | 160 | 129 | 133 | 95 | 107 |
| 8608 | 140 | 152 | 114 | 122 | 202 | 202 | 200 | 206 | 123 | 123 | 141 | 141 | 119 | 121 | 290 | 298 | 92 | 96 | 94 | 102 | 182 | 210 | 119 | 123 | 160 | 164 | 117 | 129 | 107 | 107 |
| 8924 | 152 | 152 | 120 | 120 | 200 | 202 | 200 | 206 | 127 | 127 | 151 | 151 | 121 | 121 | 310 | 318 | 96 | 96 | 94 | 102 | 154 | 178 | 123 | 131 | 164 | 164 | 129 | 129 | 95 | 101 |
| 8925 | 152 | 152 | 120 | 122 | 200 | 202 | 200 | 206 | 127 | 127 | 145 | 151 | 121 | 121 | 294 | 318 | 96 | 96 | 94 | 94 | 154 | 182 | 123 | 123 | 160 | 164 | 129 | 129 | 101 | 101 |
| 8927 | 140 | 152 | 122 | 122 | 200 | 202 | 200 | 206 | 119 | 127 | 141 | 145 | 119 | 121 | 294 | 310 | 94 | 94 | 86 | 94 | 170 | 174 | 115 | 123 | 138 | 160 | 129 | 129 | 99 | 101 |
| 8206 | 152 | 152 | 120 | 122 | 202 | 202 | 200 | 200 | 127 | 127 | 141 | 145 | 121 | 121 | 298 | 318 | 96 | 96 | 94 | 102 | 178 | 182 | 111 | 111 | 160 | 164 | 129 | 129 | 93 | 95 |
| 8609 | 140 | 140 | 114 | 114 | 202 | 204 | 200 | 202 | 123 | 127 | 145 | 151 | 121 | 121 | 310 | 310 | 92 | 92 | 94 | 102 | 178 | 178 | 119 | 119 | 160 | 160 | 129 | 129 | 95 | 95 |
| 8610 | 142 | 142 | 120 | 122 | 202 | 202 | 200 | 200 | 127 | 127 | 151 | 151 | 121 | 121 | 290 | 290 | 92 | 92 | 94 | 102 | 162 | 182 | 119 | 131 | 160 | 160 | 129 | 133 | 95 | 95 |
| 8611 | 142 | 152 | 120 | 120 | 202 | 202 | 200 | 200 | 127 | 127 | 145 | 151 | 121 | 121 | 310 | 318 | 92 | 92 | 94 | 102 | 182 | 182 | 119 | 131 | 160 | 162 | 129 | 133 | 95 | 95 |
| 8612 | 142 | 152 | 114 | 120 | 202 | 202 | 200 | 200 | 123 | 127 | 145 | 151 | 121 | 121 | 314 | 318 | 92 | 96 | 82 | 102 | 178 | 202 | 123 | 123 | 158 | 160 | 129 | 129 | 101 | 101 |
| 8613 | 142 | 152 | 120 | 120 | 202 | 202 | 198 | 200 | 123 | 127 | 151 | 151 | 121 | 121 | 318 | 318 | 92 | 92 | 94 | 102 | 182 | 182 | 119 | 123 | 160 | 160 | 129 | 133 | 93 | 95 |
| 8614 | 142 | 142 | 120 | 120 | 202 | 204 | 200 | 202 | 127 | 127 | 145 | 151 | 119 | 121 | 310 | 318 | 92 | 96 | 82 | 102 | 178 | 182 | 123 | 131 | 160 | 160 | 129 | 129 | 99 | 101 |
| 8615 | 140 | 142 | 120 | 122 | 202 | 202 | 200 | 200 | 123 | 127 | 151 | 151 | 119 | 121 | 310 | 310 | 92 | 96 | 82 | 102 | 154 | 182 | 119 | 119 | 160 | 160 | 117 | 129 | 95 | 95 |
| 8616 | 142 | 152 | 114 | 122 | 200 | 202 | 200 | 200 | 123 | 127 | 151 | 151 | 119 | 121 | 310 | 310 | 92 | 96 | 94 | 102 | 178 | 202 | 123 | 131 | 162 | 162 | 129 | 133 | 95 | 101 |
| 8617 | 152 | 152 | 120 | 122 | 202 | 202 | 200 | 200 | 127 | 129 | 145 | 151 | 121 | 121 | 302 | 318 | 92 | 96 | 94 | 102 | 154 | 154 | 111 | 131 | 160 | 164 | 129 | 129 | 99 | 101 |
| 8618 | 140 | 152 | 120 | 122 | 202 | 202 | 200 | 200 | 129 | 129 | 145 | 151 | 119 | 121 | 310 | 318 | 92 | 96 | 94 | 102 | 154 | 202 | 111 | 131 | 160 | 160 | 129 | 129 | 95 | 99 |
| 8619 | 152 | 152 | 120 | 120 | 202 | 202 | 200 | 200 | 127 | 129 | 141 | 151 | 119 | 121 | 310 | 318 | 92 | 100 | 102 | 102 | 154 | 202 | 119 | 131 | 160 | 160 | 117 | 129 | 99 | 101 |
| 8620 | 140 | 152 | 114 | 122 | 202 | 202 | 200 | 200 | 127 | 129 | 145 | 145 | 119 | 121 | 290 | 314 | 92 | 92 | 82 | 102 | 158 | 178 | 111 | 131 | 160 | 160 | 127 | 131 | 95 | 103 |
| 8621 | 142 | 152 | 120 | 120 | 202 | 202 | 200 | 202 | 123 | 129 | 145 | 151 | 121 | 121 | 310 | 310 | 92 | 92 | 82 | 82 | 154 | 178 | 119 | 131 | 160 | 160 | 117 | 129 | 101 | 101 |
| 8622 | 142 | 142 | 120 | 122 | 202 | 202 | 200 | 202 | 127 | 129 | 145 | 151 | 121 | 121 | 310 | 318 | 96 | 96 | 94 | 102 | 178 | 202 | 111 | 119 | 160 | 160 | 117 | 129 | 99 | 107 |
| 8623 | 142 | 152 | 114 | 120 | 202 | 202 | 200 | 200 | 123 | 127 | 151 | 151 | 119 | 121 | 318 | 318 | 92 | 96 | 94 | 102 | 158 | 178 | 123 | 131 | 160 | 162 | 129 | 129 | 93 | 101 |
| 8624 | 140 | 142 | 120 | 120 | 200 | 202 | 200 | 200 | 123 | 127 | 145 | 151 | 121 | 121 | 310 | 318 | 96 | 96 | 82 | 94 | 154 | 178 | 111 | 123 | 160 | 160 | 117 | 129 | 95 | 101 |
| 8625 | 140 | 140 | 114 | 120 | 200 | 202 | 200 | 200 | 127 | 127 | 145 | 145 | 113 | 121 | 302 | 302 | 96 | 96 | 94 | 102 | 162 | 178 | 123 | 123 | 160 | 160 | 131 | 131 | 101 | 101 |
| 8626 | 152 | 152 | 120 | 120 | 202 | 202 | 200 | 200 | 127 | 129 | 151 | 151 | 121 | 121 | 306 | 310 | 92 | 96 | 82 | 102 | 178 | 182 | 131 | 131 | 160 | 162 | 117 | 117 | 101 | 101 |
| 8627 | 152 | 152 | 114 | 120 | 202 | 202 | 200 | 200 | 127 | 129 | 145 | 151 | 121 | 121 | 310 | 310 | 92 | 96 | 94 | 102 | 154 | 178 | 111 | 119 | 160 | 162 | 117 | 117 | 101 | 101 |
| 8628 | 142 | 152 | 114 | 122 | 202 | 202 | 200 | 200 | 127 | 127 | 151 | 151 | 121 | 121 | 310 | 310 | 92 | 96 | 102 | 102 | 178 | 178 | 119 | 119 | 160 | 160 | 129 | 131 | 95 | 95 |
| 8629 | 142 | 142 | 120 | 122 | 200 | 202 | 200 | 200 | 123 | 127 | 145 | 151 | 121 | 121 | 310 | 318 | 96 | 96 | 82 | 102 | 154 | 178 | 123 | 123 | 160 | 160 | 129 | 133 | 95 | 101 |
| 8630 | 142 | 142 | 122 | 122 | 202 | 202 | 200 | 200 | 123 | 127 | 151 | 151 | 119 | 121 | 310 | 314 | 92 | 96 | 82 | 102 | 178 | 182 | 111 | 119 | 160 | 160 | 129 | 129 | 95 | 101 |
| 8631 | 142 | 152 | 120 | 122 | 198 | 202 | 200 | 200 | 123 | 127 | 151 | 151 | 119 | 121 | 310 | 318 | 92 | 96 | 86 | 94 | 154 | 182 | 111 | 123 | 160 | 160 | 129 | 133 | 93 | 95 |
| 8632 | 140 | 142 | 120 | 122 | 200 | 202 | 200 | 200 | 123 | 127 | 151 | 151 | 121 | 121 | 310 | 310 | 92 | 92 | 82 | 102 | 154 | 158 | 111 | 111 | 160 | 164 | 129 | 131 | 95 | 101 |
| 8852 | 152 | 152 | 120 | 122 | 198 | 198 | 200 | 200 | 127 | 127 | 151 | 157 | 121 | 121 | 314 | 318 | 92 | 92 | 94 | 102 | 154 | 178 | 123 | 131 | 160 | 164 | 129 | 131 | 93 | 93 |
| 8853 | 140 | 152 | 114 | 120 | 202 | 202 | 200 | 202 | 123 | 127 | 151 | 151 | 121 | 121 | 306 | 310 | 92 | 96 | 82 | 106 | 154 | 186 | 119 | 131 | 160 | 160 | 129 | 129 | 95 | 99 |
| 8854 | 142 | 142 | 122 | 122 | 198 | 202 | 200 | 202 | 123 | 127 | 151 | 157 | 115 | 121 | 302 | 310 | 96 | 96 | 82 | 94 | 162 | 202 | 111 | 131 | 160 | 160 | 129 | 131 | 93 | 95 |
| 8855 | 140 | 152 | 120 | 120 | 198 | 202 | 200 | 200 | 127 | 129 | 141 | 157 | 113 | 119 | 310 | 318 | 92 | 96 | 102 | 102 | 162 | 202 | 111 | 111 | 164 | 164 | 129 | 129 | 101 | 101 |
| 8856 | 140 | 140 | 120 | 122 | 198 | 198 | 200 | 202 | 127 | 127 | 151 | 157 | 119 | 119 | 302 | 318 | 92 | 96 | 94 | 102 | 182 | 202 | 111 | 111 | 160 | 160 | 129 | 129 | 101 | 107 |
| 8857 | 152 | 152 | 114 | 120 | 198 | 198 | 200 | 200 | 127 | 127 | 151 | 151 | 115 | 121 | 310 | 318 | 92 | 96 | 94 | 102 | 162 | 182 | 111 | 123 | 160 | 164 | 129 | 131 | 93 | 95 |
| 8858 | 142 | 152 | 114 | 120 | 198 | 200 | 200 | 200 | 127 | 129 | 145 | 151 | 121 | 121 | 302 | 310 | 92 | 92 | 102 | 106 | 182 | 182 | 123 | 131 | 160 | 160 | 129 | 133 | 107 | 107 |
| 8859 | 142 | 142 | 120 | 120 | 198 | 202 | 200 | 200 | 125 | 127 | 151 | 157 | 121 | 121 | 302 | 310 | 92 | 92 | 94 | 102 | 182 | 182 | 131 | 131 | 160 | 160 | 129 | 129 | 93 | 99 |
| 8860 | 142 | 152 | 114 | 114 | 198 | 200 | 200 | 200 | 127 | 127 | 141 | 151 | 119 | 121 | 302 | 302 | 96 | 100 | 102 | 102 | 154 | 202 | 111 | 131 | 160 | 160 | 129 | 129 | 95 | 95 |
| 8861 | 152 | 152 | 114 | 120 | 198 | 202 | 200 | 200 | 123 | 127 | 151 | 157 | 121 | 121 | 314 | 318 | 92 | 92 | 82 | 102 | 154 | 182 | 119 | 119 | 162 | 162 | 117 | 129 | 95 | 101 |
| 8986 | 140 | 140 | 122 | 122 | 200 | 200 | 200 | 200 | 123 | 129 | 145 | 145 | 121 | 121 | 294 | 310 | 92 | 96 | 82 | 102 | 154 | 178 | 111 | 147 | 160 | 160 | 133 | 133 | 99 | 101 |
| 5732 | 152 | 154 | 114 | 114 | 200 | 200 | 188 | 200 | 123 | 125 | 141 | 151 | 121 | 121 | 310 | 322 | 92 | 96 | 102 | 102 | 178 | 206 | 111 | 123 | 160 | 164 | 131 | 131 | 93 | 99 |
| 5733 | 140 | 142 | 114 | 114 | 200 | 200 | 200 | 200 | 123 | 123 | 145 | 147 | 115 | 119 | 294 | 310 | 92 | 96 | 94 | 102 | 154 | 182 | 111 | 123 | 160 | 164 | 127 | 131 | 101 | 101 |
| 5737 | 142 | 142 | 120 | 120 | 200 | 200 | 200 | 200 | 125 | 125 | 141 | 145 | 115 | 121 | 290 | 310 | 96 | 96 | 94 | 102 | 154 | 178 | 131 | 131 | 160 | 160 | 117 | 127 | 97 | 99 |
| 5787 | 142 | 150 | 120 | 120 | 200 | 202 | 188 | 188 | 123 | 123 | 145 | 145 | 119 | 121 | 310 | 310 | 96 | 96 | 94 | 102 | 154 | 174 | 123 | 123 | 160 | 162 | 117 | 129 | 95 | 95 |
| 5814 | 142 | 142 | 114 | 120 | 200 | 200 | 188 | 188 | 123 | 123 | 145 | 145 | 119 | 119 | 286 | 286 | 92 | 92 | 94 | 94 | 154 | 154 | 123 | 123 | 0 | 0 | 127 | 127 | 91 | 91 |
| 6355 | 140 | 152 | 120 | 120 | 200 | 200 | 188 | 200 | 123 | 123 | 145 | 145 | 119 | 121 | 286 | 318 | 92 | 96 | 94 | 102 | 154 | 170 | 119 | 119 | 164 | 164 | 117 | 117 | 101 | 107 |
| 7021 | 140 | 142 | 120 | 122 | 200 | 202 | 200 | 200 | 123 | 123 | 145 | 145 | 121 | 121 | 298 | 318 | 96 | 96 | 82 | 102 | 154 | 154 | 119 | 123 | 160 | 162 | 127 | 133 | 99 | 99 |
| 8322 | 142 | 142 | 120 | 120 | 200 | 200 | 200 | 200 | 123 | 127 | 145 | 147 | 119 | 119 | 286 | 294 | 92 | 92 | 94 | 102 | 174 | 174 | 123 | 123 | 160 | 160 | 117 | 117 | 97 | 101 |
| 8457 | 142 | 142 | 120 | 120 | 200 | 202 | 200 | 200 | 123 | 127 | 141 | 145 | 115 | 121 | 310 | 318 | 92 | 96 | 102 | 102 | 154 | 202 | 123 | 123 | 164 | 164 | 117 | 117 | 101 | 101 |
| 8919 | 140 | 142 | 114 | 122 | 200 | 200 | 194 | 200 | 127 | 127 | 141 | 145 | 115 | 121 | 294 | 314 | 92 | 96 | 94 | 102 | 154 | 154 | 123 | 123 | 160 | 164 | 117 | 133 | 95 | 99 |
| 8920 | 140 | 142 | 122 | 122 | 200 | 200 | 188 | 194 | 123 | 127 | 141 | 151 | 119 | 119 | 290 | 310 | 92 | 96 | 102 | 106 | 150 | 178 | 123 | 123 | 160 | 160 | 127 | 133 | 107 | 107 |
| 8921 | 140 | 150 | 114 | 120 | 200 | 200 | 194 | 194 | 119 | 123 | 137 | 145 | 115 | 121 | 282 | 318 | 96 | 96 | 94 | 106 | 150 | 150 | 123 | 123 | 160 | 160 | 133 | 133 | 99 | 101 |
| 8922 | 136 | 152 | 114 | 122 | 202 | 202 | 200 | 206 | 123 | 123 | 137 | 145 | 115 | 121 | 282 | 310 | 92 | 96 | 94 | 102 | 154 | 154 | 123 | 123 | 160 | 160 | 131 | 133 | 99 | 107 |
| 8923 | 142 | 150 | 114 | 114 | 200 | 202 | 200 | 206 | 123 | 127 | 145 | 145 | 115 | 115 | 314 | 314 | 96 | 96 | 94 | 94 | 150 | 162 | 123 | 123 | 160 | 160 | 131 | 133 | 93 | 95 |
| 9225 | 142 | 142 | 120 | 122 | 202 | 202 | 198 | 200 | 129 | 129 | 145 | 157 | 119 | 121 | 310 | 310 | 92 | 92 | 94 | 102 | 154 | 178 | 119 | 123 | 160 | 160 | 131 | 131 | 99 | 107 |
| 7869 | 140 | 152 | 114 | 120 | 202 | 202 | 188 | 200 | 123 | 123 | 145 | 157 | 119 | 119 | 290 | 318 | 92 | 96 | 94 | 102 | 178 | 182 | 111 | 131 | 160 | 164 | 127 | 131 | 93 | 107 |
| 8267 | 140 | 140 | 114 | 114 | 202 | 202 | 200 | 200 | 123 | 125 | 141 | 151 | 115 | 119 | 294 | 310 | 92 | 96 | 94 | 102 | 154 | 154 | 123 | 123 | 160 | 164 | 127 | 129 | 95 | 95 |
| 8887 | 142 | 144 | 120 | 120 | 202 | 202 | 176 | 188 | 123 | 123 | 141 | 145 | 115 | 119 | 290 | 294 | 92 | 96 | 102 | 102 | 174 | 178 | 119 | 123 | 158 | 160 | 127 | 127 | 93 | 95 |
| 8888 | 142 | 142 | 114 | 120 | 202 | 202 | 200 | 206 | 123 | 123 | 145 | 151 | 115 | 119 | 290 | 310 | 92 | 92 | 94 | 102 | 202 | 218 | 123 | 135 | 160 | 160 | 117 | 117 | 95 | 99 |
| 8960 | 152 | 152 | 114 | 120 | 200 | 200 | 200 | 200 | 123 | 127 | 151 | 151 | 121 | 121 | 290 | 318 | 90 | 90 | 76 | 82 | 154 | 154 | 111 | 123 | 160 | 160 | 129 | 129 | 95 | 95 |
| 8961 | 152 | 152 | 114 | 114 | 200 | 202 | 200 | 200 | 123 | 127 | 145 | 151 | 119 | 119 | 298 | 310 | 96 | 96 | 76 | 82 | 150 | 154 | 111 | 111 | 160 | 160 | 129 | 129 | 95 | 95 |
| 8962 | 152 | 152 | 120 | 122 | 200 | 200 | 200 | 200 | 123 | 127 | 145 | 151 | 119 | 121 | 310 | 310 | 90 | 96 | 82 | 102 | 150 | 154 | 111 | 111 | 160 | 160 | 129 | 129 | 101 | 101 |
| 8963 | 152 | 152 | 114 | 120 | 200 | 200 | 200 | 200 | 123 | 127 | 151 | 151 | 119 | 121 | 290 | 318 | 90 | 96 | 82 | 102 | 154 | 154 | 111 | 123 | 160 | 160 | 129 | 129 | 95 | 95 |
| 8964 | 140 | 152 | 114 | 120 | 198 | 200 | 200 | 200 | 123 | 127 | 151 | 151 | 119 | 121 | 290 | 310 | 96 | 96 | 82 | 102 | 150 | 154 | 111 | 111 | 160 | 160 | 129 | 129 | 95 | 101 |
| 8965 | 140 | 140 | 120 | 122 | 198 | 204 | 200 | 200 | 123 | 123 | 145 | 151 | 121 | 121 | 298 | 310 | 92 | 96 | 82 | 94 | 154 | 206 | 111 | 111 | 160 | 160 | 129 | 129 | 95 | 101 |
| 8967 | 140 | 152 | 120 | 122 | 198 | 204 | 200 | 200 | 127 | 127 | 151 | 151 | 119 | 121 | 298 | 310 | 92 | 96 | 82 | 94 | 150 | 154 | 111 | 123 | 160 | 160 | 129 | 129 | 95 | 101 |
| 8968 | 140 | 152 | 114 | 120 | 204 | 204 | 200 | 200 | 123 | 123 | 151 | 151 | 121 | 121 | 298 | 298 | 92 | 96 | 82 | 94 | 146 | 154 | 111 | 123 | 160 | 160 | 129 | 129 | 95 | 101 |
| 8971 | 140 | 152 | 114 | 120 | 198 | 202 | 200 | 200 | 123 | 123 | 141 | 151 | 121 | 121 | 298 | 298 | 96 | 96 | 82 | 82 | 150 | 150 | 111 | 111 | 158 | 160 | 129 | 129 | 95 | 99 |
| 9064 | 140 | 140 | 114 | 114 | 198 | 202 | 200 | 200 | 123 | 127 | 151 | 151 | 121 | 121 | 298 | 306 | 90 | 90 | 82 | 102 | 154 | 154 | 111 | 111 | 160 | 160 | 129 | 129 | 93 | 101 |
| 9065 | 152 | 152 | 120 | 120 | 200 | 200 | 200 | 200 | 123 | 127 | 145 | 151 | 121 | 121 | 290 | 314 | 96 | 96 | 76 | 82 | 146 | 154 | 111 | 123 | 160 | 164 | 129 | 129 | 101 | 101 |
| 8972 | 140 | 140 | 120 | 122 | 200 | 200 | 200 | 200 | 123 | 127 | 145 | 145 | 115 | 121 | 314 | 314 | 96 | 96 | 82 | 102 | 154 | 170 | 123 | 123 | 160 | 160 | 117 | 117 | 99 | 101 |
| 8973 | 140 | 142 | 122 | 122 | 200 | 200 | 198 | 200 | 127 | 127 | 145 | 145 | 115 | 119 | 314 | 314 | 96 | 96 | 94 | 102 | 170 | 170 | 123 | 155 | 138 | 138 | 117 | 117 | 95 | 101 |
| 8976 | 140 | 140 | 122 | 122 | 200 | 200 | 200 | 200 | 123 | 127 | 145 | 145 | 119 | 119 | 290 | 314 | 96 | 96 | 86 | 94 | 158 | 170 | 123 | 155 | 164 | 164 | 117 | 117 | 101 | 101 |
| 8977 | 140 | 140 | 120 | 122 | 200 | 200 | 200 | 200 | 123 | 123 | 145 | 145 | 119 | 119 | 314 | 314 | 96 | 96 | 86 | 94 | 170 | 170 | 123 | 155 | 164 | 164 | 117 | 129 | 93 | 101 |
| 8978 | 140 | 140 | 122 | 122 | 200 | 200 | 200 | 200 | 123 | 123 | 145 | 145 | 119 | 119 | 290 | 314 | 96 | 96 | 94 | 102 | 158 | 170 | 123 | 155 | 138 | 164 | 129 | 137 | 99 | 99 |
| 8979 | 140 | 142 | 122 | 122 | 200 | 200 | 200 | 200 | 123 | 127 | 145 | 145 | 119 | 121 | 314 | 314 | 96 | 96 | 86 | 94 | 170 | 182 | 123 | 123 | 160 | 164 | 117 | 117 | 93 | 95 |
| 8980 | 140 | 140 | 122 | 122 | 200 | 200 | 200 | 200 | 119 | 127 | 145 | 145 | 115 | 119 | 314 | 314 | 96 | 96 | 86 | 94 | 170 | 182 | 111 | 111 | 164 | 164 | 117 | 137 | 93 | 101 |
| 8981 | 140 | 140 | 122 | 122 | 200 | 200 | 200 | 200 | 127 | 127 | 145 | 145 | 119 | 119 | 314 | 314 | 96 | 96 | 86 | 94 | 158 | 158 | 111 | 111 | 164 | 164 | 117 | 117 | 93 | 101 |
| 8982 | 140 | 140 | 120 | 122 | 200 | 200 | 200 | 200 | 123 | 127 | 145 | 145 | 119 | 119 | 290 | 314 | 96 | 96 | 94 | 102 | 158 | 170 | 111 | 155 | 164 | 164 | 117 | 129 | 93 | 95 |
| 8985 | 140 | 140 | 122 | 122 | 200 | 200 | 200 | 200 | 123 | 127 | 145 | 145 | 119 | 119 | 290 | 314 | 92 | 92 | 82 | 86 | 158 | 170 | 119 | 155 | 164 | 164 | 117 | 137 | 99 | 99 |
|  |  |  |  |  |  |  |  |  |  |  |  |  |  |  |  |  |  |  |  |  |  |  |  |  |  |  |  |  |  |  |

**Table C. *Per locus* genetic diversity in 97 samples of golden jackal.** *A* ­- number of alleles; *H*_O_ - heterozygosity observed; *H*_E_ - heterozygosity expected; HWE - *P-*values for HWE exact test for heterozygote deficiency/excess (significant deviation from HWE indicated by *); *F*_IS_ - fixation index; * - statistical significance of *F*_IS_ (Bonferroni corrected *P*-value, based on 300 randomizations, at α = 0.05 was 0.0033).

| Parameter | CPH4 | CPH5 | CPH8 | CPH12 | CPH6 | CPH9 | CPH22 | FH2004 | FH2088 | FH2096 | FH2137 | FH2140 | CXX.213 | C09.250 | C20.253 |
| --- | --- | --- | --- | --- | --- | --- | --- | --- | --- | --- | --- | --- | --- | --- | --- |
| *A* | 7 | 3 | 4 | 7 | 5 | 6 | 4 | 11 | 5 | 6 | 14 | 8 | 7 | 6 | 9 |
| *H*_O_ | 0.505 | 0.546 | 0.340 | 0.258 | 0.567 | 0.536 | 0.423 | 0.711 | 0.433 | 0.845 | 0.722 | 0.546 | 0.365 | 0.433 | 0.619 |
| *H*_E_ | 0.686 | 0.628 | 0.611 | 0.336 | 0.619 | 0.636 | 0.534 | 0.812 | 0.549 | 0.680 | 0.824 | 0.729 | 0.501 | 0.685 | 0.767 |
| HWE | * |  | * | * |  |  |  | * | * | * | * | * | * | * | * |
| *F*_IS_ | 0.261* | 0.143 | 0.444* | 0.250* | 0.070 | 0.166 | 0.224 | 0.123 | 0.207 | -0.233 | 0.134 | 0.261* | 0.275* | 0.362* | 0.201* |

**Table D. *Per region* genetic diversity, estimated based on polymorphisms in 15 microsatellite loci.** SEE - south-eastern Europe; CAU - Caucasus; BAL - Baltics; GRE-P - Greece, Peloponnese; GRE-S - Greece, Samos Island. *N* - sample size; *A* ­- number of alleles; *R* - allelic richness; *H*_O_ - heterozygosity observed; *H*_E_ - heterozygosity expected; HWE - *P-*values for HWE exact test for heterozygote deficiency/excess (significant deviation from HWE indicated by *); *F*_IS_ - fixation index. Significant *F*_IS_ values marked by * (Bonferroni corrected *P*-value, based on 300 randomizations, at *α* = 0.05 was 0.0067).

| Parameter | CPH4 | CPH5 | CPH8 | CPH12 | CPH6 | CPH9 | CPH22 | FH2004 | FH2088 | FH2096 | FH2137 | FH2140 | CXX. 213 | C09.250 | C20.253 | Mean |
| --- | --- | --- | --- | --- | --- | --- | --- | --- | --- | --- | --- | --- | --- | --- | --- | --- |
|  | SEE (*N* = 57) | | | | | | | | | | | | | | | |
| *A* | 3 | 3 | 4 | 4 | 5 | 4 | 4 | 8 | 5 | 5 | 11 | 6 | 7 | 5 | 7 | 5.40 |
| *R* | 2.92 | 2.81 | 2.83 | 2.12 | 3.11 | 3.09 | 2.23 | 4.33 | 2.50 | 3.15 | 4.82 | 3.91 | 2.89 | 3.37 | 4.23 | 3.22 |
| *H*_O_ | 0.544 | 0.596 | 0.404 | 0.246 | 0.614 | 0.579 | 0.368 | 0.737 | 0.474 | 0.842 | 0.772 | 0.614 | 0.404 | 0.474 | 0.614 | 0.552 |
| *H*_E_ | 0.646 | 0.586 | 0.480 | 0.277 | 0.594 | 0.565 | 0.366 | 0.734 | 0.550 | 0.633 | 0.795 | 0.746 | 0.454 | 0.602 | 0.752 | 0.585 |
| HWE |  |  |  |  |  |  |  |  | * | * |  |  |  | * | * | * |
| *F*_IS_ | 0.167 | -0.009 | 0.167 | 0.121 | -0.024 | -0.016 | 0.003 | 0.005 | 0.147 | -0.322 | 0.037 | 0.186 | 0.119 | 0.221 | 0.192 | 0.066 |
|  | CAU (*N* = 14) | | | | | | | | | | | | | | | |
| *A* | 6 | 3 | 2 | 4 | 4 | 5 | 3 | 9 | 2 | 4 | 9 | 4 | 3 | 5 | 7 | 4.67 |
| *R* | 4.17 | 2.91 | 1.95 | 3.42 | 3.06 | 3.70 | 2.98 | 6.09 | 2.00 | 2.95 | 5.14 | 2.94 | 2.61 | 4.19 | 5.43 | 3.57 |
| *H*_O_ | 0.714 | 0.357 | 0.286 | 0.429 | 0.429 | 0.643 | 0.571 | 0.786 | 0.500 | 0.714 | 0.571 | 0.214 | 0.385 | 0.571 | 0.571 | 0.516 |
| *H*_E_ | 0.679 | 0.625 | 0.337 | 0.625 | 0.528 | 0.584 | 0.661 | 0.842 | 0.477 | 0.594 | 0.735 | 0.416 | 0.494 | 0.755 | 0.819 | 0.611 |
| HWE |  |  |  |  |  |  |  |  |  |  |  | * |  |  |  |  |
| *F*_IS_ | -0.016 | 0.458 | 0.188 | 0.347 | 0.224 | -0.064 | 0.171 | 0.103 | -0.011 | -0.166 | 0.257 | 0.513 | 0.259 | 0.278 | 0.335 | 0.192* |
|  | BAL (*N* = 5) | | | | | | | | | | | | | | | |
| *A* | 4 | 3 | 1 | 5 | 3 | 4 | 3 | 4 | 2 | 2 | 6 | 5 | 3 | 4 | 4 | 3.53 |
| *R* | 4.00 | 3.00 | 1.00 | 5.00 | 3.00 | 4.00 | 3.00 | 4.00 | 2.00 | 2.00 | 6.00 | 5.00 | 3.00 | 4.00 | 4.00 | 3.53 |
| *H*_O_ | 0.400 | 0.600 | 0.000 | 0.800 | 0.200 | 1.000 | 0.800 | 0.800 | 0.600 | 0.800 | 0.800 | 0.800 | 0.600 | 0.400 | 0.800 | 0.400 |
| *H*_E_ | 0.640 | 0.580 | 0.000 | 0.680 | 0.460 | 0.720 | 0.540 | 0.700 | 0.420 | 0.480 | 0.780 | 0.680 | 0.460 | 0.700 | 0.720 | 0.640 |
| HWE |  |  |  |  |  |  |  |  |  |  |  |  |  |  |  |  |
| *F*_IS_ | 0.467 | 0.077 | NA | -0.067 | 0.636 | -0.290 | -0.391 | -0.032 | -0.333 | -0.600 | 0.086 | -0.067 | -0.200 | 0.515 | 0.000 | 0.013 |
|  | GRE-P (*N* = 11) | | | | | | | | | | | | | | | |
| *A* | 2 | 3 | 4 | 1 | 2 | 3 | 2 | 6 | 3 | 4 | 4 | 2 | 3 | 1 | 4 | 2.933 |
| *R* | 2.00 | 2.86 | 3.76 | 1.00 | 2.00 | 2.39 | 1.99 | 4.54 | 2.85 | 3.65 | 3.16 | 1.97 | 1.91 | 1.00 | 2.91 | 2.53 |
| *H*_O_ | 0.364 | 0.727 | 0.545 | 0.000 | 0.636 | 0.455 | 0.364 | 0.727 | 0.455 | 0.909 | 0.636 | 0.455 | 0.182 | 0.000 | 0.545 | 0.467 |
| *H*_E_ | 0.463 | 0.607 | 0.690 | 0.000 | 0.483 | 0.368 | 0.397 | 0.748 | 0.558 | 0.632 | 0.566 | 0.351 | 0.169 | 0.000 | 0.579 | 0.441 |
| HWE |  |  |  |  |  |  |  |  |  |  |  |  |  |  |  |  |
| *F*_IS_ | 0.259 | -0.151 | 0.255 | NA | -0.273 | -0.190 | 0.130 | 0.075 | 0.231 | -0.399 | -0.077 | -0.250 | -0.026 | NA | 0.104 | -0.011 |
|  | GRE-S (*N* = 10) | | | | | | | | | | | | | | | |
| *A* | 2 | 2 | 1 | 2 | 3 | 1 | 3 | 2 | 2 | 4 | 4 | 4 | 3 | 3 | 4 | 2.667 |
| *R* | 1.76 | 1.90 | 1.00 | 1.50 | 2.50 | 1.00 | 2.66 | 1.96 | 1.76 | 3.71 | 3.26 | 3.48 | 2.79 | 2.79 | 3.86 | 2.40 |
| *H*_O_ | 0.200 | 0.300 | 0.000 | 0.100 | 0.600 | 0.000 | 0.400 | 0.400 | 0.000 | 1.000 | 0.700 | 0.600 | 0.200 | 0.500 | 0.700 | 0.380 |
| *H*_E_ | 0.180 | 0.255 | 0.000 | 0.095 | 0.545 | 0.000 | 0.405 | 0.320 | 0.180 | 0.700 | 0.595 | 0.685 | 0.465 | 0.465 | 0.730 | 0.375 |
| HWE |  |  |  |  |  |  |  |  |  |  |  |  |  |  |  |  |
| *F*_IS_ | -0.059 | -0.125 | NA | 0.000 | -0.049 | NA | 0.065 | -0.200 | 1.000 | -0.385 | -0.125 | 0.176 | 0.604 | -0.023 | 0.094 | 0.038 |
